# Anticancer Compounds Based on Isatin-Derivatives: Strategies to Ameliorate Selectivity and Efficiency

**DOI:** 10.3389/fmolb.2020.627272

**Published:** 2021-02-04

**Authors:** Raphael Enoque Ferraz de Paiva, Eduardo Guimarães Vieira, Daniel Rodrigues da Silva, Camila Anchau Wegermann, Ana Maria Costa Ferreira

**Affiliations:** Departamento de Química Fundamental, Instituto de Química, Universidade de São Paulo, São Paulo, Brazil

**Keywords:** oxindoles, isatin, anticancer activity, metallodrugs, mechanisms of action, improvement strategies

## Abstract

In this review we compare and discuss results of compounds already reported as anticancer agents based on isatin-derivatives, metalated as well as non-metallated. Isatin compounds can be obtained from plants, marine animals, and is also found in human fluids as a metabolite of amino acids. Its derivatives include imines, hydrazones, thiosemicarbazones, among others, already focused on numerous anticancer studies. Some of them have entered in pre-clinical and clinical tests as antiangiogenic compounds or inhibitors of crucial proteins. As free ligands or coordinated to metal ions, such isatin derivatives showed promising antiproliferative properties against different cancer cells, targeting different biomolecules or organelles. Binding to metal ions usually improves its biological properties, indicating a modulation by the metal and by the ligand in a synergistic process. They also reveal diverse mechanisms of action, being able of binding DNA, generating reactive species that cause oxidative damage, and inhibiting selected proteins. Strategies used to improve the efficiency and selectivity of these compounds comprise structural modification of the ligands, metalation with different ions, syntheses of mononuclear and dinuclear species, and use of inserted or anchored compounds in selected drug delivery systems.

## Introduction and Scope of this Work

**FIGURE 1:**
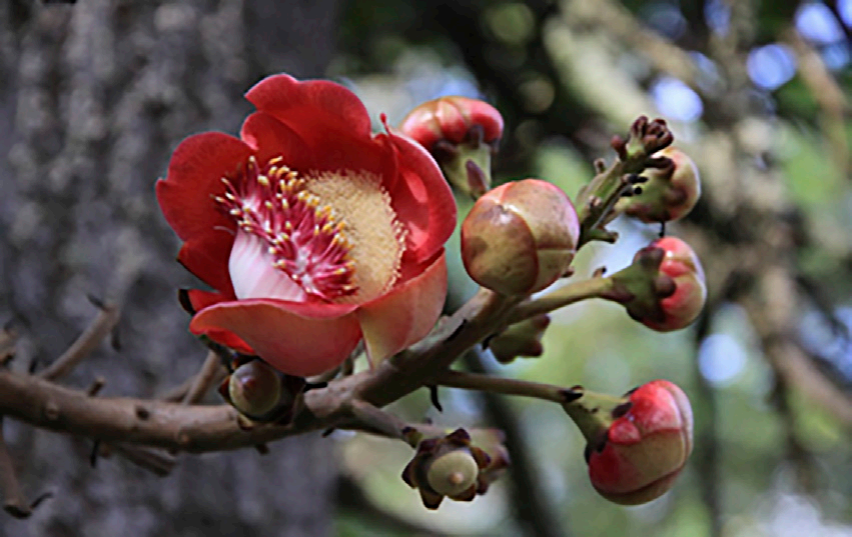
A vibrant dark red flower of Couroupita guianensis (Abricó-de-macaco) tree, photographed at the Rio de Janeiro Botanical Garden (Pacheco de Oliveira, 2011).

Isatin (1H-indol-2,3-dione) is a natural alkaloid extracted as a red-orange powder from plants of the Isatis genus (Figure 1), found around the world in *Isatis tinctoria* (Europe and China) (Zhou and Qu, 2011), *Couroupita guianensis Aubl* (Central America and Amazon region) (Bergman et al., 1985), *Melochia tomentosa* (United States, Mexico) and *Boronia koniamboensis* (New Caledonia) species (Da Silva et al., 2001; Bayly et al., 2015). Those plants contain indolic compounds that have shown medicinal properties, being already used as anti-inflammatory, and antineoplastic agents. Isatin is also found in secretions of the Bufo frog (Europe, North Africa, and Asia), and in the Australian mollusc Dicathais orbita (Esmaeelian et al., 2014). Additionally, it is an endogenous compound in humans, being detected as a metabolite of tryptophan or epinephrine, and largely disseminated in the central nervous system (CNS) (Kerzarea and Khedekar, 2016).

As a versatile molecule, isatin is the precursor of a huge number of derivatives, containing the oxindole moiety and presenting a wide range of biological and pharmacological properties. Numerous reviews on its synthesis (Silva, 2013; Vine et al., 2013) and possible applications have been reported in the literature (Pandeya et al., 2005; Varun et al., 2019; Nath et al., 2020). Isatin-derivatives have been developed as anticonvulsant (Cheke et al., 2018; Saini et al., 2016; Shridar et al., 2002), anti-stress and anxiogenic, (Medvedev et al., 2005), antiviral (Zhang et al., 2015), antimicrobial (Bharathi Dileepan et al., 2018) antitubercular (Vintonyak et al., 2010; Xu et al., 2017), antimalarial (Raj et al., 2014), antifungal (Rodríguez-Argüelles et al., 2009), antibacterial (Guo, 2019) and particularly as antitumor agents (Harris, 2007), among a wide range of activities, with a peculiar comprehensive~diversity in their structures. Some derivatives were designed and already tested in clinical trials against cancer, and a few others have been approved as anticancer drugs (Izzedine et al., 2007; Prakash et al., 2012). Others were reported more recently as possibly active in metabolic diseases (diabetes) (Rahim et al., 2015) and as inhibitors of cholinesterase and β-amyloid aggregation (Marques et al., 2020).

**FIGURE 2:**
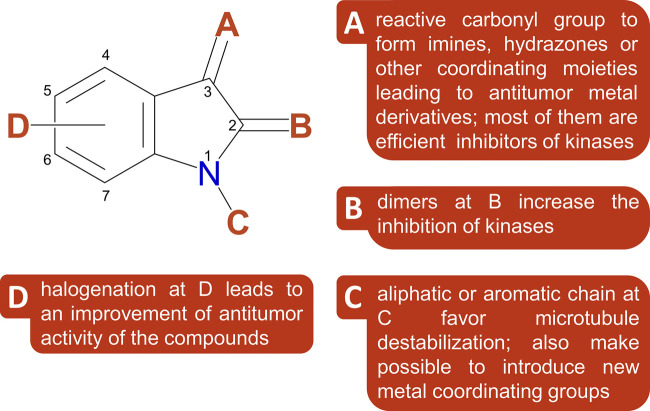
A structure-reactivity scheme of isatin-derivatives, identifying the substituents that favor specific activities (based on Vine et al., 2013).

Herein, we intend to report a broad spectrum of biological and medicinal properties of isatin-based compounds, with emphasis in their anticancer activities. Many isatin derivatives have been synthesized on the purpose of achieving effective and selective medicinal agents, with different substituents at any site of its core structure. A diversity of substituents at N1, C3 and any C in the benzene ring provided a modulation in their properties, and positions at C3 (substituents A) and C5, C6 or C7 (substituents D) in the aromatic ring seems to be the most encouraging ones (see Figure 2).

Therefore, imines, hydrazones, thiosemicarbazones, oximes, spiro-oxindoles, among other compounds were prepared, characterized, and had their biological properties evaluated along the last decades. Many reviews have been published, exploring different aspects of the chemistry of such group of compounds, especially those related to pharmacological and medicinal aspects. Nevertheless, the field remains extensively explored, with many advances achieved in recent years.

**FIGURE 3:**
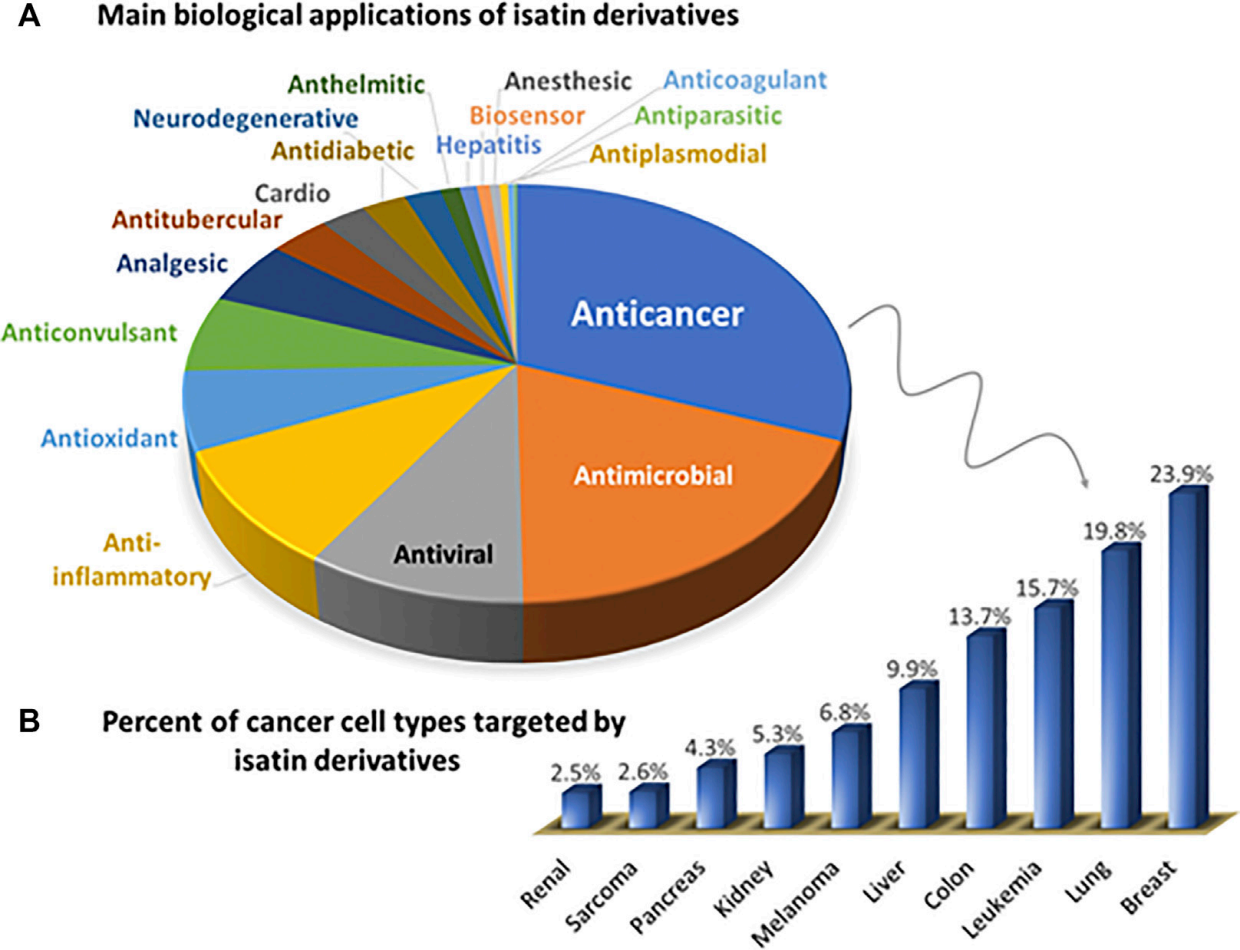
**(A)** Distribution of biological applications of isatin-derivatives reported in the literature (using keywords: isatin-type of biological application). **(B)** Further details on the Anticancer group shown in **(A)**, highlighting the types of cancer targeted by isatin-derivatives, in percentage (keywords: isatin-cancer-type of cancer). All publication data was obtained from SciFinder, covering the period from 1951 until September 2020.

A recent search on SciFinder, performed by us in September 2020, pointed to more than ten thousand articles and reviews published since 1951 containing the keyword “isatin”. The pie chart shown in Figure 3A shows the entries found by combining “isatin” with “type of biological application.” (example: anticoagulant, or biosensor). The bar chart (Figure 3B) highlights the main types of cancer studied, using the keywords “isatin,” “anticancer” and “type of cancer” (example: lung, or breast). In Figure 3B, 9% of all the publications dealing with anticancer investigations explore kinases as an important target for this class of compounds.

Despite the predominance of biological applications, other areas also deserved high interest, as catalysis, dye compounds, nanocomposites, polymers, sensor (not considered in this work). The wide range of applications of isatin derivatives is certainly related to its underlying versatility that allows the construction of diverse structures suitable for a specific reactivity or a chemical property of interest.

Among the biological investigations, anticancer and antimicrobial compounds were the most extensively explored. However, the range of beneficial properties described for this class of compounds also includes antiviral, anti-inflammatory, and antioxidant as the major applications. Breast (21%), lung (17%) and leukemia (14%) tumor cells are the most studied since they correspond to the more recurrent types of cancer according to the World Health Organization (WHO).

Based on this search, we discuss herein some of the studies developed exploring mostly the possible modes of action of isatin-derivatives toward cancer, and some strategies used in the literature and in our laboratory to improve their antitumoral properties.

## Oxindoles as a Class of Compounds of Pharmacological Interest

Isatin has emerged as a promising scaffold for the design and development of new medicinal agents. However, oxindoles as a class of compounds containing a bicyclic core structure with a benzene ring fused to a pyrrole ring and having a carbonyl group at second position, are natural products ubiquitously found in plants, bacteria, invertebrates, and mammals. Further, naturally occurring oxindole alkaloids inspired a plethora of new synthesized derivatives motivated by their pharmacological activities (Kaur et al., 2016; Marchese et al., 2020). Particularly, 2-oxindole which are spiro-fused to other cyclic frameworks (Singh and Desta, 2012) merited a large interest of researchers because of their occurrence in varied natural products and high diversity of bioactivity, especially as antiviral agents (Ye et al., 2016). Also, they constitute a challenge for synthetic organic chemists, demanding specific strategies and asymmetric methods to achieve peculiar structures (Rios, 2012). In Section *Isatin-Derivatives as Anticancer Agents*, some relevant examples of oxindole structures are discussed. Additionally, introduction of imine, amine, hydrazone or thiosemicarbazone moieties at A position (see Figure 2) leads to metal binding sites, providing additional possibilities of structural changes.

**FIGURE 4:**
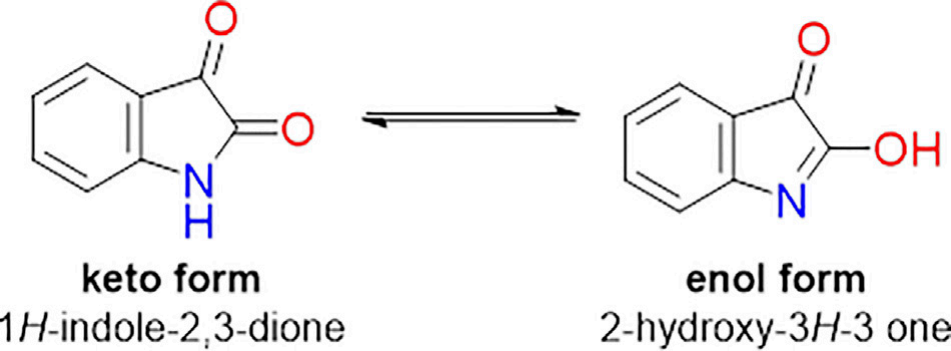
Tautomeric equilibrium for free isatin.

This class of compounds exhibit in solution a characteristic tautomeric equilibrium, as exemplified for isatin (Figure 4), which is dependent on the temperature and polarity of the solvent. For the synthesis of derivatives, these factors, in addition to the control of pH, can be explored to obtain preferentially one of these tautomers.

The incorporation of good coordinating groups around the isatin ring allowed the metal complex formation of such molecules, which led to very stable transition metal complexes, also obtained as keto- as well as enol-species. Consequently cationic, or neutral metal complexes species were designed and prepared, and had its properties evaluated in comparison to the corresponding free ligand compounds.

## Strategies to Improve Efficiency or Selectivity of Isatin-Derivatives

### Structural Changes

Usually, structural changes in organic compounds with the insertion of electron accepting or electron donating substituents, more lipophilic or hydrophilic groups, delocalization of electron density, can improve its properties for selected purposes. According to our search, a diversity of coordinating groups was introduced in the oxindole core, at different points of it, modulating the reactivity of isatin-derivatives as antiproliferative agents. Particularly, N donor groups (imines, amines, hydrazines, hydrazones, oximes, thiosemicarbazones) inserted in the isatin ring core were valuable and endorsed further studies, as discussed in Section *Isatin-Derivatives as Anticancer Agents*.

Recently, hybridization of isatin with other anticancer pharmacophores as imines, azoles, quinolines, quinazolines, sulfonamides, coumarins, was reviewed, as another approach of providing attractive scaffolds for new anticancer agents, more efficient and less toxic (Ding et al., 2020).

Also, the use of prodrugs at the aim of reducing side effects of therapeutics has been recently reported (Karnthaler-Benbakka et al., 2019). A conjugate of a cleavable dipeptide with an approved drug, Sunitinib, showed slightly reduced antiproliferative results against different tumors cells. However, the hydrolytic lability of the prodrug was pointed out as a shortcoming.

### Metal Complex Formation

Another common approach to get better activity of organic compounds is to bind them to metal ions, introducing charges, providing a more rigid conformation around the metal centre, and therefore influencing their interactions with biomolecules. On the other hand, an adequate ligand can improve the metal uptake by cells and organelles, in a synergistic process that facilitates interactions and possible damage to biomolecules. Further, ligands can remarkably modify the reduction potential of metals, with consequent enhancement or inhibition of their activity. In this way, modification of ligand structural features, with the introduction of additional coordinating groups in appropriate sites of the molecule can be extremely helpful. Design of bi-, tri- or polydentate ligands, depending on the chosen metal ion and corresponding biological target, can be crucial to achieve more efficient and selective compounds. A substantial number of metal complexes containing isatin-based ligands has been described as antitumor compounds, especially manganese(II), cobalt(II), nickel(II), and copper(II) complexes, with different coordination geometries and reactivities, that are discussed in Section *Anticancer Metal Complexes with Isatin-Containing Ligands*.

### Drug Delivery Systems

Additionally, another useful strategy to improve the biological activity of these derivatives is to combine the most active compounds with suitable drug delivery systems that can protect them from off-site metabolic degradation. These systems can act as carriers of the compounds into the cell, and to selected organelles, acting as adjuvant in antiproliferative processes, in reactive intermediates generation *in situ*, and enhancement of selectivity. Besides, the matrices also provide a modified delivery of the drugs in fluids or inside the cells, helping to maintain an adequate concentration of the active compounds along the time.

Free isatin-derivatives, especially those already approved as antitumor drug, and few metal complexes of isatin-containing ligands have been combined with drug delivery systems for antitumoral applications. These systems introduced higher reactivity and/or selectivity, in addition to a better control of its delivery, as discussed in Section *Carriers for Modified Delivery of Isatin-Derivatives*.

## Isatin-Derivatives as Anticancer Agents

Among the many pharmacological or medicinal uses of isatin and isatin-derivatives, their antiproliferative and antitumor properties deserve special attention. The facility in derivatizing the isatin ring in different points (see Figure 2), inserting a variety of functions including coordinating groups lead to a huge number of studies with prevalence of anticancer investigations, as shown in Figure 3. Consequently, some isatin-derivatives entered in clinical tests (in different phases) and a few ones became approved anticancer drugs (see discussion and references cited in Section Hydrazines and Hydrazones).

### Diversity of New Organic Isatin-Derivatives Investigated

Isatin and correlated compounds have been described in the literature since the 50’ and 60.’ Nevertheless, in the last two decades an increased interest was observed probably motivated by their possible applications in different areas. Diverse compounds containing the oxindole moiety have been investigated by pharmaceutical industries or research groups, being the most cited imines, hydrazones, oximes, thiosemicarbazones, spiro-compounds, as discussed below in Sections *Imines* to *Miscellaneous Functionalization*. Those studies comprehended natural compounds, from plants and animals, as well as synthetic compounds, and strongly promoted a developing knowledge of the chemistry and the biology of this kind of compounds.

#### Imines

Imines, as an ubiquitous moiety in biological medium and a versatile pharmacophore already tested, have been extensively investigated as antitumor compounds, especially di-imines or Schiff bases (Kajal et al., 2013; Tadele and Tsega, 2019). Aromatic imines usually interact with DNA by π stacking between base pairs, causing severe disturbance in the helixes, and inducing apoptosis.

Imines containing the isatin scaffold were less investigated as metal-free ligands than the corresponding metal complexes (see also Section *Anticancer Metal Complexes with Isatin-Containing Ligands*) (Cerchiaro and Ferreira, 2006; Tadele and Tsega, 2019). However, many investigations indicated also good properties of the non-metallated compounds against proliferation of tumor cells (Vine et al., 2013; Varun et al, 2019).

A recent study reported one-pot, multicomponent syntheses of a series of isatin-based imidazole compounds, active toward inflammation and cancer (Kumar et al., 2020). These compounds were able to inhibit *in vitro* the enzymes cyclooxygenase-2 (COX-2), an enzyme responsible for inflammation, and phosphoinositide-3 kinase (PI3K), a crucial enzyme in breast cancer. Experimental data were supported by molecular docking and structure-activity relationship (SAR), that helped to screen the most effective imidazole derivatives.

Isatin-based hybrids incorporating different anticancer pharmacophores, as coumarin, quinoline, quinazoline, azole, and imines have been reviewed recently (Ding et al., 2020). Imine fragments are usually inserted at point A in the isatin ring (see Figure 2), providing a good site for coordination of metal ions, and exhibiting antiproliferative activity against different cancer cells (HepG2, HCT-116, CACO, and MCF-7), with IC_50_ values in the range <10–100 µM comparable or better than 5-fluorouracil, but not superior to that of doxorubicin (IC_50_ = 4.56–8.29 μM). The introduction of benzyl group into N-1 position of isatin moiety seems to lead to more active derivatives, as attested by SAR studies (Farooq et al., 2018). Also, incorporation of halogen atoms into C-5, C-6 or C-7 position of isatin core probably improve its activity. Brominated isatin- or indirubin-derivatives extracted from marine organisms, as molluscs and gastropods, have been investigated, exhibiting good activity against tumor cells (Esmaeelian et al., 2014). Some of them were verified to be inhibitors of kinases, particularly cyclin dependent kinase CDK2, a key target for this kind of compounds (Choi et al., 2010).

In another study, it has been demonstrated that oxindolimines can stabilize the tumor suppressor gene p53, in human bone osteosarcoma epithelial U2OS-pLV cell line (Davidovich et al., 2015).

Multiple compounds can be defined as imine derivatives, containing multiple functional groups. Among them, hydrazones and thiosemicarbazones containing the C=N bond conjugated to different groups, which gives each them intrinsic and varied properties (Yousef et al., 2020).

#### Hydrazines and Hydrazones

Hydrazines and hydrazones play an important role in chemical and biological environment due to their structural versatility and reactivity. They have been also used as synthetic intermediates to produce a wide number of molecular hybrids, not only applied as potential pharmaceuticals, but also as industrial agents (Scott et al., 2010; Elder et al., 2011).

**FIGURE 5:**
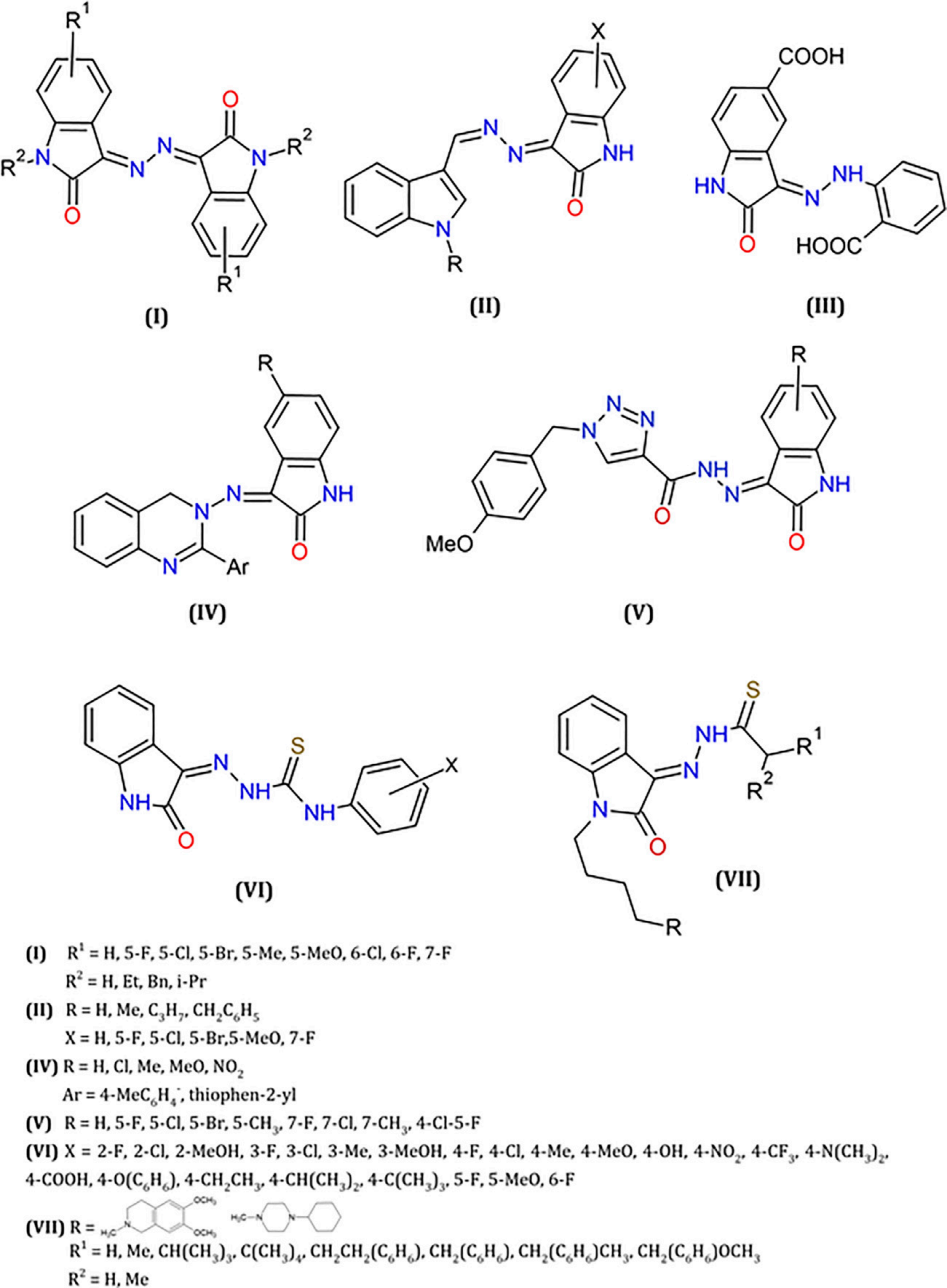
Some examples of isatin hydrazines or isatin thiosemicarbazones reported as antitumor agents.

A set of hybrids of isatin with hydrazines and hydrazones moieties have been extensively described in the literature, comprehending natural products (Le Goff and Ouazzani, 2014) and synthetic derivatives (El-Faham et al., 2015) due to their biological and, especially, their anticancer properties. These studies include different derivatives, as coumarins (Nasr et al., 2014), triazines (Hamama et al., 2018), pyrazole-based heterocycles (Ismail and El-sayed, 2019), exhibiting good activity versus diverse tumor cells (colon, leukemia, breast, kidney). Some of these hydrazine- and hydrazone derivatives of isatin are shown in Figure 5.

Particularly, hydrazine moieties were used as a linker to provide the construction of bis-isatin compounds with wide structural variety, and biological activities (Ibrahim et al., 2016). In an interesting study, a series of symmetrical bis-Schiff base derivatives were obtained by condensation of isatins, natural or synthetic, with hydrazine (Liang et al., 2014) (I, Figure 5). Evaluation of their in vitro antitumor activities indicated significant results, against various cancer cell lines (A549, lung adenocarcinoma epithelial cell; Hela, epithelial carcinoma cell; HepG2, liver hepatocellular cell; U251, glioma cell; SGC-7901, human gastric carcinoma cells). For the most active compound (3,3′-(hydrazine-1,2-diylidene)bis(5-methylindolin-2-one), IC_50_ values in the range 4–13 µM were determined, and further *in vivo* studies indicated a substantial inhibition of tumor growth in mice.

Several isatin-hydrazine hybrid structures presented good antitumor activities against cancer cell lines such as breast, lung and leukemia (Abdel-Aziz et al., 2012; Azizian et al., 2012; Dweedar et al., 2014; Eldehna et al., 2015; Prakash et al., 2018). Derivatives of [(3-indolylmethylene)hydrazono]indolin-2-one (II, Figure 5), for example, were tested against breast cell lines (MCF-7, MDA-MB-231). They induce a G2/M arrest and activate the caspase 3 and caspase 9 pathways in response to the production of reactive oxygen species (ROS). They also suppress the expression of the anti-apoptotic protein Bcl-2 and increase the expression of the pro-apoptotic protein BAX (Fares et al., 2015; Eldehna et al., 2017; Eldehna et al., 2018).

Isatin-hydrazones (Lawrence et al., 2008; Scott et al., 2010; Nicholas et al., 2015) were evaluated comparatively to the analogue oxindole NSC 117199, known inhibitor of the protein tyrosine phosphatase Shp2 (IC_50_ = 47 μM). These molecules, including compound III in Figure 5 have shown to be promising inhibitors of Shp2 and Shp1 (IC_50_ = 0.8 μM against Shp2).

Some isatin-quinazoline hydrazones derivatives and analogues (IV, Figure 5) were also tested comparatively to CFM-1, (Z)-5(5-bromo-7-methyl-2-oxoindolin-3-ylidene)-2-thioxothiazolidin4-one, known as CARP-1 Functional Mimetic (CFMs) and perinuclear phosphoprotein, CARP-1/CCAR1 inhibitor (Puliyappadamba et al., 2011). These compounds display good inhibition activity against several types of cancer cell lines (Alafeefy et al., 2015).

Isatin-triazole hydrazones (V, Figure 5), were also described as anticancer agents as well as potent inhibitors of Microtubule affinity-regulating kinase 4 (MARK4), known to induce proliferation and migration of cells in breast cancer, also associated with hepatocellular cancer (Naz et al., 2013). In both cases, apoptotic process is induced by the generation of ROS (Aneja et al., 2019).

These compounds were also tested in the inhibition of selected proteins and enzymes implicated in cancer. The ability of hydrazino-isatin-based benzenesulfonamides in the inhibition of cyclin-dependent kinase 2 (CDK2) were previously reported, exhibiting antiproliferative activity against different cancer cell lines HT29, MDAMB468, RKO and SW620. These studies were supported by molecular modeling and crystallography that helped to understand the interaction between the tested compounds and the CDK2 protein (Bramson et al., 2001). Recently, these compounds were also reported as inhibitors of human carbonic anhydrase isoenzyme IX (hCA IX), exhibiting a better activity than the standard CA inhibitor acetazolamide (AAZ) used as reference (Hamama et al., 2018).

#### Thiosemicarbazones

Thiosemicarbazones are compounds characterized by component RC = NNC = SNR’ which distinguishes them from the synthetic point of view as versatile structures. Besides, these compounds have substantial scientific interest due their biological properties as anticancer agents.

A library of Isatin-β-thiosemicarbazones derivatives (VI, Figure 5) was synthesized and it was found that these compounds can block P-gp (P-glycoprotein) expression from cervical adenocarcinoma cell lines (KB-V1), and present good cytotoxicity against parental Hela from cervical cancer cell line (KB-3-1). In this study, the prediction of selectivity for multi-drug-resistant (MDR1) genes and its cytotoxicity was supported by quantitative structure-activity relationships 3D-QSAR analysis (Hall et al., 2009; Hall et al., 2011).

The treatment of C57BL/6 female mice with another class of isatin-thiosemicarbazones (VII, Figure 5), led to a reduction in tumor size inoculated subcutaneously. Importantly, this class of compounds were also described as multitarget agents against σ2 receptors and capable to interact with P-gp protein (Pati et al., 2015; Pati et al., 2018).

For metal complexes with isatin-thiosemicarbazones ligands, see Section *Isatin-Hydrazone*, *-Thiocarbazine and -Thiosemicarbazone Metal Complexes*.

#### Miscellaneous Functionalization

Not only isatin but also indirubin and related compounds were used as precursor in these studies. *In vitro* antiproliferation studies of isatin-derivatives, obtained from the condensation of isatin or indirubin with active methylene heterocycles, showed also high cytotoxicity against different cancer cells, as glioma, breast, and especially against some resistant cells to apoptosis as melanoma (SK-MEL-28) and esophageal (OE21) (Evdokimov et al., 2016).

Novel isatin-3-oxime-based hydroxamic acids have been designed and investigated for the inhibition of histone-H3 and histone-H4 deacetylation, since histone deacetylases (HDACs) inhibitors were described as promising agent for cancer treatment (Nam et al., 2013). Those derivatives, with an oxime group at A, and a halogen substituent at D position in isatin ring, besides a hydroxamic acid chain at C, exhibited strong cytotoxicity, with low IC_50_ values (<10 µM) toward different tumor cells (SW620, colon cancer; MCF-7, breast cancer; PC3, prostate cancer; AsPC-1, pancreatic cancer; NCI-H460, lung cancer).

A series of triazole tethered isatin-coumarin hybrids were prepared and tested against diverse human tumor cells. The most active compounds were also evaluated as tubulin polymerization inhibitors, exhibiting IC_50_ values of a few µM. Molecular modeling studies revealed that their cytotoxicity depends on the isatin-substituents, and the length of carbon-bridge connecting isatin moiety with the triazole ring (Singh et al., 2017).

Additionally, a series of moxifloxacin-isatin hybrids tethered via 1,2,3-triazole at the N_1_ of isatin core (see Figure 2) were synthesized, and had their *in vitro* anticancer activities tested against a panel of cancer cell lines, including liver (HepG2), breast (MCF-7), analogous doxorubicin resistant (MCF-7/DOX), prostate (DU-145) and analogous multidrug-resistant (MDR DU-145) cells. The most active hybrid in the series showed IC_50_ values in the range 32 to 77 μM, more active than Vorinostat (or suberoylanilide hydroxamic acid, SAHA) with IC_50_ >100 μM, used for comparison (Yang et al., 2020).

### Derivatives Clinically Approved or Undergoing Clinical Trials

There is a long way for a compound to become an approved drug. From synthesis to the evaluation of main targets and mechanisms of action, and subsequently a series of clinical tests demand many efforts and a huge financial support. Even with this large selection, some approved drugs still have undesirable side effects for patients (Ansari et al., 2010). In the case of oxindoles, despite of these difficulties, several derivatives entered clinical tests and a few ones were approved as an anticancer drug.

Particularly, the most promising isatin-derivatives, Sunitinib, and Toceranib, have been clinically approved against tumors. Sunitinib inhibits the catalytic activity of kinases in the phosphorylation of other proteins, by reversible binding to the ATP binding site of these target proteins (Roskoski, 2007). Further, this compound showed good oral bioavailability, and demonstrated efficacy in preclinical tumor models. Consequently, Sunitinib was approved by the FDA (US Food and Drug Administration) for the treatment of gastrointestinal stromal tumors and advanced renal cell carcinoma (RCC) in 2006, for a rare type of pancreatic cancer in 2011, and as adjuvant agent for treatment of patients at high risk for recurrent renal carcinoma, in 2017. It is currently being clinically tested in conjunction with new immunotherapeutics (Rizzo and Porta, 2017). Toceranib, a similar compound to Sunitinib, was approved for the treatment of the canine mast cell tumor (MCT) in 2009 by FDA. It acts as a selective inhibitor of some receptor tyrosine kinases (RTKs), presenting antiproliferative activity in endothelial cells, and a potential cell cycle arresting effect inducing tumor cells apoptosis *in vivo* (London et al., 2003; London et al., 2009).

The studies involving Sunitinib were extremely important for the development of other biologically active isatin derivatives. Until the 2000s, according with data obtained from Scifinder platform, only 32 articles had been published with isatin derivatives with antitumor properties. This number increased by almost 70% (2001–2010) and more than 300% (2011–2020) with the first publications of Sunitinib and its subsequent approval by FDA (see Figure 3).

Recently, it was reported a Cathepsin B-cleavable prodrug of Sunitinib as a strategy aimed at reducing its side effects (Karnthaler-Benbakka et al., 2019). The conjugate with a Phe-Lys cleavable dipeptide, and a self-immolative linker that suffers a 1,6-elimination. Cathepsin B cleavage assay confirmed the desired enzymatic activation of the compound. Despite showing a comparable antiproliferative potency to Sunitinib against diverse cell lines (HCT116, Caki-1 and RU-MH), the conjugate showed undesirable hydrolytic lability.

**FIGURE 6:**
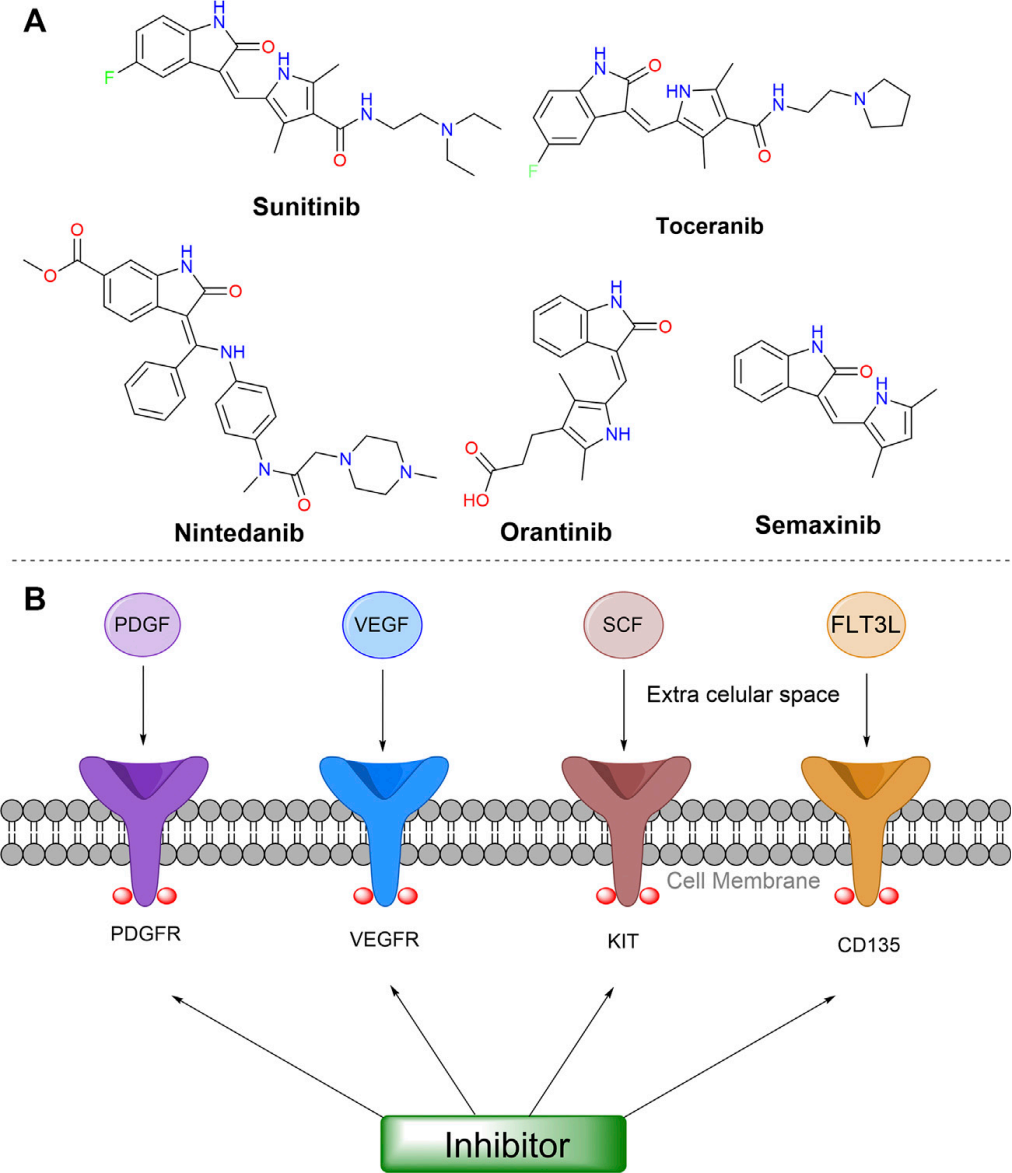
**(A)** Structures of isatin-derived compounds in clinical trials or already approved. **(B)** Some targets involved in general mechanism of action of these compounds. Abbreviations: VEGF, vascular endothelial growth factor; VEGFR, vascular endothelial growth factor receptors; PDGF, platelet-derived growth factor; PDGFR, platelet-derived growth factor receptors; KIT, stem cell factor receptor; SCF, stem-cell factor; CD135, cluster of differentiation antigen; FLT3L, FMS-like tyrosine kinase 3 ligand.

Some other derivatives, Nintedanib, Semaxinib, and Orantinib, showing interesting structural similarities are currently undergoing clinical tests, for colorectal tumor, ovarian cancer, hepatocellular carcinoma, mesothelioma, prostate cancer, glioblastoma, renal cell carcinoma, endometrial cancer, among others. These compounds have a pyrrole or an imidazole moiety as an A-substituent in the same position in the isatin ring, as shown in Figure 2. Those specific substituents at isatin were reported to inhibit receptor tyrosine kinases (Vine et al., 2009), including platelet-derived growth factor receptors (PDGFRα/PDGFRβ), vascular endothelial growth factor receptor (VEGFR-1/VEGFR-2), stem-cell factor receptor (KIT), and cluster of differentiation antigen (CD135), which are somehow linked to the growth of tumors and angiogenesis (Eisen et al., 2015; McCormack, 2015). Additionally, these drugs showed the ability to reduce or stop tumor growth, by modulation of cell growth, proliferation, survival, and migration (Le Tourneau et al., 2007), as shown in Figure 6 in a simplified way.

A huge number of registered clinical tests were carried out (more than 900 for Sunitinib) toward a diversity of cancers. A few of them completed phase 3 tests and have become approved drugs by FDA; some others are still in clinical trials at different stages. Among these trials, some deserve highlights. Sunitinib entered in phase 3 studies in combination with other anticancer agents, such as Docetaxel, Capecitabine and Paclitaxel (Robert et al., 2011; Crown et al., 2013). More recently, Nintedanib was tested free or combined with *Docetaxel*, *Paclitaxel* and Carboplatin (Van Cutsem et al., 2018; Barra et al., 2019). Free Orantinib entered in phase 1 and phase 2 studies, as well as in combination with transcatheter arterial chemoembolisation up to phase 3 (Kanai et al., 2011; Kudo et al., 2018). Free Semaxinib was tested at phase 2, and at phase 3 in combination with 5-Fluorouracil, *Leucovorin and Irinotecan* in patients with metastatic colorectal cancer (Lockhart et al, 2006).

Table 1 summarizes some relevant clinical studies on the family of Sunitinib and related oxindole-derivatives that are used as anticancer agents, also highlighting the types of cancer treated by each compound (ClinicalTrials.gov database, U.S. National Library of Medicine).

**TABLE 1:** Level of investigation on some compounds already approved or under clinical trials, according to ClinicalTrials.gov database (U.S. National Library of Medicine).

Compound	Level of investigation
Sunitinib	Approved for gastrointestinal stromal tumors and advanced renal cell carcinoma in 2006, by FDA.
	Approved for a rare type of pancreatic cancer in 2011, by FDA.
	Approved as adjuvant agent for treatment of patients at high risk for recurrent renal carcinoma in 2017, by FDA.
	Phase 3 completed tested in patients with breast cancer in combination with Docetaxel, Capecitabine, and Paclitaxel.
Toceranib	Approved for the treatment of the canine mast cell tumor in 2009, by FDA
Nintedanib	Phase 3 completed tested in patients with refractory metastatic colorectal cancer in 2018.
	Phase 3 completed tested in combination with Paclitaxel and Carboplatin in first line treatment of ovarian cancer in 2018.
	Phase 3 completed tested in combination with Docetaxel in 2nd line non-small cell lung cancer in 2018.
Orantinib	Phase 1/2 completed study for advanced hepatocellular carcinoma in 2011.
	Phase 3 study combined with transcatheter arterial chemoembolization in patients with unresectable hepatocellular carcinoma in 2017.
Semaxinib	Phase 3 completed study in combination with 5-Fluorouracil, Leucovorin, and Irinotecan for metastatic colorectal cancer in 2004.
	Phase 2 completed study in patients with persistent or recurrent cervical cancer in 2003.
	Phase 2 completed study in patients with advanced or recurrent head and neck cancer in 2009.

However, some of these candidates to anticancer drugs exhibited adverse effects, as low effectiveness in some studies, diarrhea, hypertension, vomiting, hand – foot syndrome, and neutropenia (Ansari et al., 2010). An undesirable cardiotoxicity induced by Sunitinib has been detected along its clinical tests, motivating toxicological mechanism studies and cardioprotective therapies (Yang and Bu, 2016). Therefore, further investigations in the development of correlated compounds were needed, and many research groups in addition to pharmaceutical industries embraced this challenge.

## Anticancer Metal Complexes with Isatin-Containing Ligands

As described in numerous investigations in the literature, metal-complexes of isatin derivatives show even better properties than the free ligands regarding their reactivity in biological medium, especially as antiproliferative and pro-apoptotic agents. Many types of ligands were used as scaffold, coordinated to different metal ions, for developing novel and more efficient metallodrugs. Some of them are discussed here.

### Coordination Compounds

#### Isatin-Hydrazone, -Thiocarbazine and -Thiosemicarbazone Metal Complexes

**FIGURE 7:**
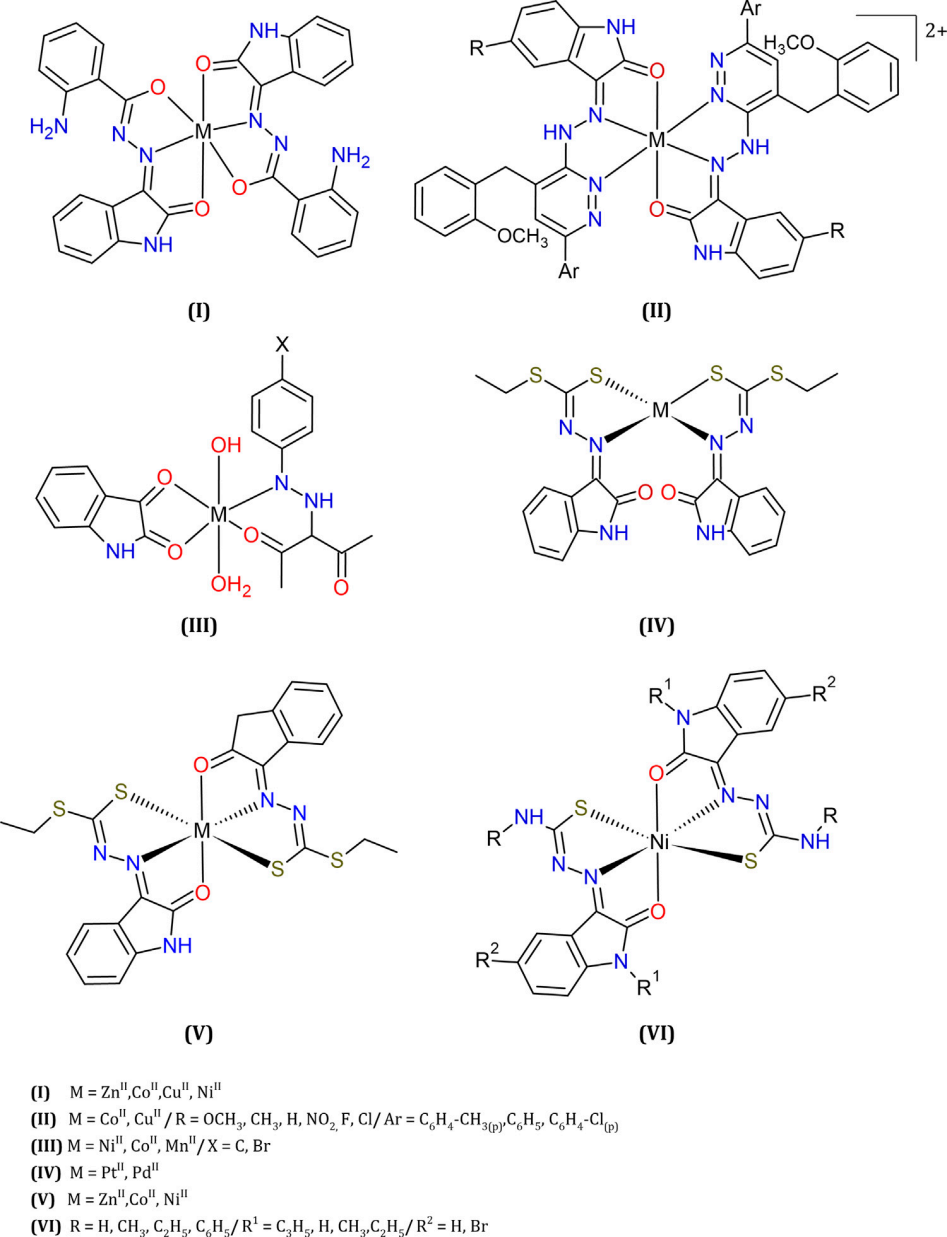
Some examples of metal complexes with isatin-derivatives: hydrazone, thiocarbazine or thiosemicarbazone, reported as antitumor agents.

Many hydrazones derived from isatin were used in complex formation reactions and gave rise to stable metal complexes with significant antiproliferative properties, especially complexes of divalent ions such as manganese(II), cobalt(II), nickel(II), copper(II) and zinc(II) (see Figure 7).

Complexes of a tridentate ligand (L = isatinyl-2-aminobenzoylhydrazone) with Cu(II) or Co(II) exhibited better cytotoxicity toward Ehrlich Ascites Carcinoma cells when compared to the analogous Zn(II) or Ni(II) complexes (I, Figure 7), or the free ligand (Hunoor et al., 2015).

Similar results were described for metal ions coordinated to isatin-hydrazine pyrazine derivatives (II, Figure 7), where Cu(II) and Co(II) complexes showed higher cytotoxicity than the free Lewis bases, toward different tumor cells (HCT116, MCF7, and HeLa (Kandile et al., 2012).

Good cytotoxicity results were also found against several tumor cell lines for complexes of general formula [ML′L(OH)(OH_2_)], where M = Ni(II), Co(II), or Mn(II); L = isatin; and L′ = 3-(arylhydrazono)acetylacetone derivatives. (III, Figure 7). Further, lower IC_50_ values determined in the range nM were usually obtained for the metal compounds in comparison to the corresponding non-coordinated ligands (Osman et al., 2014).

Recently, dithiocarbazate ligands based on isatin and coordinated to different square-planar (IV, Figure 7) or octahedral (V, Figure 7) metal ions were reported (Ali et al., 2011; Ali et al., 2012; Yekke-Ghasemi et al., 2020). These compounds IV and V had their structures solved by single crystal X-ray diffraction. The cytotoxicity of each compound, in comparison to the metal-free ligand, was evaluated against a panel of three cell lines (the tumorigenic HeLa and MCF-7, and non-tumorigenic CHO). The ligand itself was highly cytotoxic, and coordination to these divalent cations increased its cytotoxicity in all cases, but for Zn(II). Against the tumorigenic cell lines, the Co(II) (V, Figure 7) emerged as the most cytotoxic.

Nickel(II) bis(isatin thiosemicarbazone) also showed good activities against several cancer cell lines (VI, Figure 7). The results showed that the treatment of myeloma cell lines (IM-9) with this complex induces the apoptosis, the cell cycle arrest in G0/G1 phase and the cleavage of plasmid DNA (Rodríguez-Argüelles et al., 1999; Haribabu et al., 2015; Balachandran et al., 2018).

Copper(II) thiosemicarbazones of isatin and other carbonyl compounds were recently compared, showing better toxicity than the metal-free ligands, toward different tumor cell lines, and inhibition of topoisomerase II (Singh et al., 2020).

#### Oxindolimines as Ligands for Essential Metals

**FIGURE 8:**
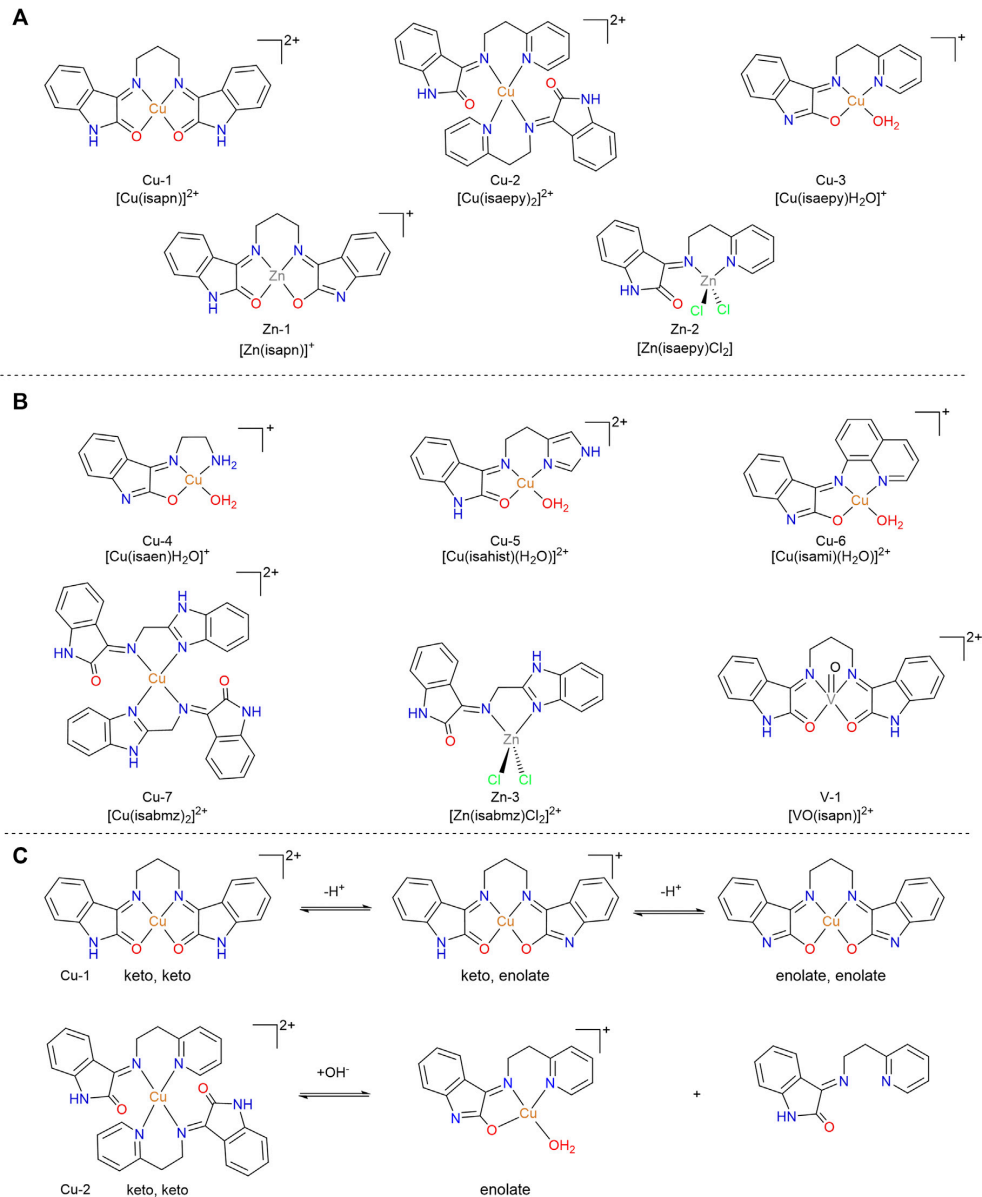
**(A)** Flagship isatin-containing copper(II) complexes developed by our group as potential metallodrugs, and their zinc(II) analogues. **(B)** Other examples of isatin-containing metallodrugs (of copper, zinc, or vanadium) explored in this review. **(C)** Keto-enolic and deprotonation equilibria for compound Cu-1 and Cu-2, illustrated as representative compounds in the series.

Our group has extensively explored isatin-containing ligands for the development of antiproliferative metallodrugs based on essential metals. Examples of these compounds are shown in Figure 8. Inspired by oxindole-derivatives that have already entered in pre-clinical and clinical tests (Lane et al., 2001), we designed, isolated, and characterized by spectroscopic techniques a series of oxindolimines. The ligands were obtained by condensation reaction of isatin with appropriate amines or di-imines (Vigato and Tamburini 2004; Vigato et al., 2007), and subsequently were metallated and isolated as corresponding metal complexes (Cerchiaro et al., 2005; Da Silveira et al, 2008), whose properties as antiproliferative agents toward different tumor cells (neuroblastomas, melanomas, sarcomas, cervicals) were determined (Filomeni et al., 2007 and 2009; Dario et al., 2019; Mamian-López et al., 2021). In all our investigations, the metal complex was more active than the corresponding free ligand. In the literature, similar results were verified with other types of ligands, attesting the efficiency of such strategy (Gou et al., 2017; Singh et al., 2020).

Similarly to the precursor isatin, all these complexes exhibit keto-enol equilibria that influence their behavior and speciation under physiological conditions (See Figure 8C). Many of them were isolated and characterized in solid state as one of the tautomeric forms, depending on the pH adjusted during the synthesis. In solution, both forms are present and usually characterized by EPR (copper species) or NMR (zinc species) spectroscopies, besides conductometry and mass spectrometry (ESI-MS). As an example, Cu-2 was isolated as a brownish powder with the ligand in the keto form, with a M:L 1:2 ratio at pH 5 (Cerchiaro et al., 2005), and alternately Cu-3 was isolated with the ligand in an enolate form, with a M:L 1:1 ratio, under basic conditions (Da Silveira et al, 2008).

##### Apoptotic Mechanism and Crucial Targets

The antiproliferative activities of compounds Cu-1 to Cu-6 was first reported on human neuroblastoma (SH-SY5Y) and promonocytic (U937) tumorigenic cell, using the Trypan blue cell counting assay (Cerchiaro et al., 2005). Compounds Cu-1, Cu-2 and Cu-6 significantly decreased viable cells count in both cell lines, while Cu-4 and Cu-5 had no effect even at concentrations as high as 50 µM, after 48 h incubation. Using cell cytometry, compound Cu-2 was shown to induce G2/M arrest in SH-SY5Y cells, while Cu-1 induced G1 phase arrest (Cerchiaro et al., 2005).

The pro-apoptotic activity of the more active compounds Cu-1 and Cu-2 were then evaluated in depth in a follow up study (Filomeni et al., 2007). Both compounds were found to trigger apoptosis in SH-SY5Y cells via the mitochondrial pathway, as determined by Western blots of total protein extracts analyzed for detection of p53 and p21, and by flow cytometry. Western blot was also used to evaluate pro- and active caspase-9, pro-caspase 3 and PARP, and apoptotic activity (sub-G1) was also evaluated in SH-SY5Y cells in a co-incubation experiments with the pancaspase inhibitor zVAD-fmk, with or without Cu-1 and Cu-2. The extent of apoptosis was also found to directly correlate with the kinetics of copper uptake (as determined by atomic absorption). This can be clearly observed for Cu-2, which enters cells more efficiently and specifically damages nuclei and mitochondria. Cu-1 was found to be less permeable to the cell membrane, but induces a widespread oxidative stress as demonstrated by determination of intracellular ROS by flow cytometry. A biological endpoint observed as consequence of ROS production was the oxidation of proteins and lipids, as determined by Western blot of carbonyl groups and a colorimetric assay for lipid peroxidation. Overexpression of Cu,Zn-SOD was identified, as means to partially counteracts cell death. Interestingly, retinoic acid-mediated differentiation was found to completely rescues cells from apoptosis induced by both Cu-1 and Cu-2. The activation of JNK- and Akt-mediated phosphorylation pathways has been found to be not functional for apoptosis induction. Moreover, it was demonstrated that both p53-dependent and -independent apoptotic pathways are responsible for the cytotoxicity of Cu-1 and Cu-2. These results are of great interest because functional p53 is frequently lost in human tumorigenesis.

Importantly, the apoptotic response observed for both Cu-1 and Cu-2 was found to be a copper-mediated event (Filomeni et al., 2007), which is responsible for inducing nuclear and mitochondrial dysfunction. This became clear as apoptosis response diminished significantly when a zinc analogous to Cu-2 was tested, or when, incubation of Cu-2 was carried out in the presence of trien (triethylenetetramine), a good copper chelator (Filomeni et al., 2007). With this observation, this study represented a pioneer contribution to the field addressing the unequivocal role and importance of both the metal center (copper) and the unique chemical characteristics of the isatin-imine ligand (delocalized lipophilic cation) in the apoptotic effect induced by this class of metallodrugs. Copper(II) is capable of catalyzing one-electron redox cycle reactions producing ROS, while isatin-imine acts not only as a carrier for the redox-active metal ion across cellular membranes but also responds to negative transmembrane potentials (relevant for mitochondrial accumulation). Interestingly, these studies also pointed to Cu-1 as a more efficient ROS producer, and Cu-2 as a more toxic and permeating compound (Filomeni et al., 2007).

These intriguing observations stimulated our interest for studying in detail the underlying mechanism of mitochondrial-related toxicity of compound Cu-2 (Filomeni et al., 2009). When looking at isolated mitochondria from mouse liver, it was demonstrated that Cu-2 increases by 60% NADH-dependent oxygen consumption, which is associated with ROS production, in an ADP-independent manner. The zinc(II) analog to Cu-3 had no effect. Therefore, to assess whether Cu-2-mediated NADH oxidation could be responsible for ROS generation, mitochondria were incubated with Cu-2 in experimental buffer containing glutamate/malate and NADH in the presence of 0.5 U catalase. The addition of catalase halved oxygen consumption, indicating that Cu-2 dissipates NADH-deriving reducing equivalents to form O_2_/H_2_O_2_. The uncoupling mitochondrial properties of compound Cu-2 were also determined *in vitro*, and further confirmed in cell experiments (Filomeni et al., 2009).

**FIGURE 9:**
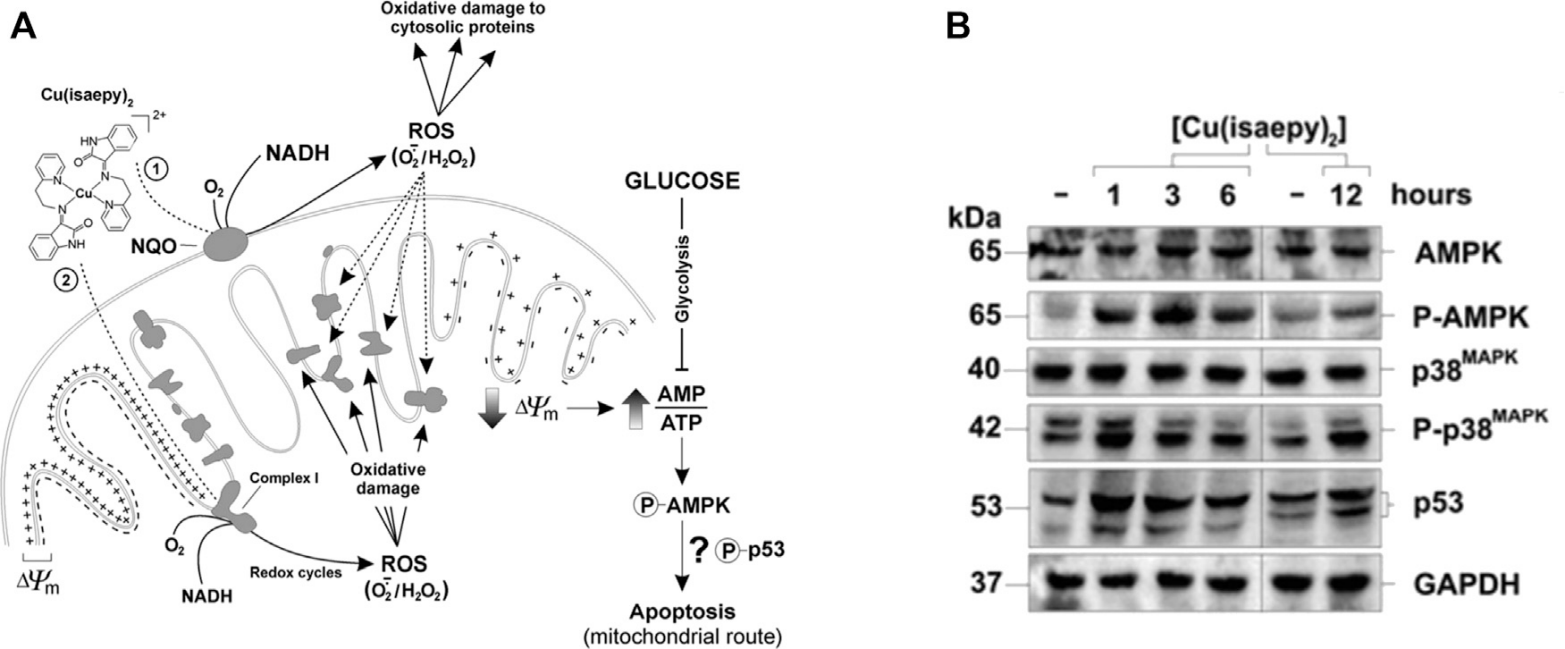
Proposed mechanism of action. **(A)** Schematic representation of the effects of Cu-2 in neuroblastoma (SH-SY5Y) cells. Cu-2 is proposed to react with NADH mainly by means of mitochondrial NADH:quinone oxidoreductases (NQO) at the outer membrane (pathway 2), but also through Complex I (pathway 1). The resulting copper-catalyzed redox cycles lead to ROS generation and consequently to a widespread oxidative damage to proteins. Membrane polarization is lost and ATP decreases, triggering the phospho-activation of AMPK, and finally leading to the mitochondrial pathway of apoptosis (Filomeni et al., 2009). **(B)** Phospho-AMPK and phospho-p38 MAPK, as well as p53, rapidly increased as soon as 1 h after Cu-2 addition to SH-SY5Y cells (Filomeni et al., 2011).

Using SH-SY5Y cells treated with Cu-2, it was possible to observe: 1) an early loss of mitochondrial transmembrane potential; 2i) a decrease in the expression levels of respiratory complex components and 3) a significant adenosine triphosphate (ATP) decrease. Overall, data indicated that Cu-2 behaves as delocalized lipophilic cation and induces mitochondrial ROS production. This event results in mitochondrial dysfunction and ATP decrease, which in turn triggers AMPK-dependent apoptosis. In terms of glucose dependency, it was found that: 1) mitochondrial membrane potential drop is tightly associates with a rapid decrease of ATP levels and insensitive to glucose supplementation; 2) glucose addition to culture media prevents or, at least, strongly delays Cu-2-mediated cell death and 3) cervix carcinoma HeLa and gastric adenocarcinoma (AGS) cells, which are known to rely most of their energy production upon mitochondrial oxidative phosphorylation, are much more susceptible toward the toxic effects of Cu-2. Also, these studies reported on AMPK (AMP-activated protein kinase) inhibition as part of a cascade of events triggered by oxidative stress leading to Cu-2-induced apoptosis (Figure 9A), and led to the hypothesis that a molecular link between AMPK and p53 and a functional synergism between them occurs in apoptosis (Filomeni et al., 2009).

The understanding of AMPK as part of the apoptosis-inducing mechanism of Cu-2 was further explored (Filomeni et al., 2011). It was found that p38 MAPK (p38 mitogen-activated protein kinase) is the molecular link in the phosphorylation cascade connecting AMPK to p53, thereby delineating an AMPK/p38 MAPK/p53 signaling axis as the principal route controlling Cu-2-induced apoptosis, with AMPK being the upstream sensor, p38 MAPK the mediator and p53 the final executioner of the cell death program (Figure 9B). Energy deficiency was found to be profoundly implicated in Cu-2-mediated toxicity, as fuel supplies (glucose in particular) counteracted Cu-2-induced apoptosis and AMPK/p38MAPK/p53 activation.

Aiming at capitalizing on this mechanism of action, it was evaluated the combination of low doses of Cu-2 with 3-bromopyruvate (3BrPA), a potent glycolytic inhibitor, which was effective and selective in inducing apoptosis in neuroblastoma cells without any significant toxicity towards differentiated primary cortical neurons.

**FIGURE 10:**
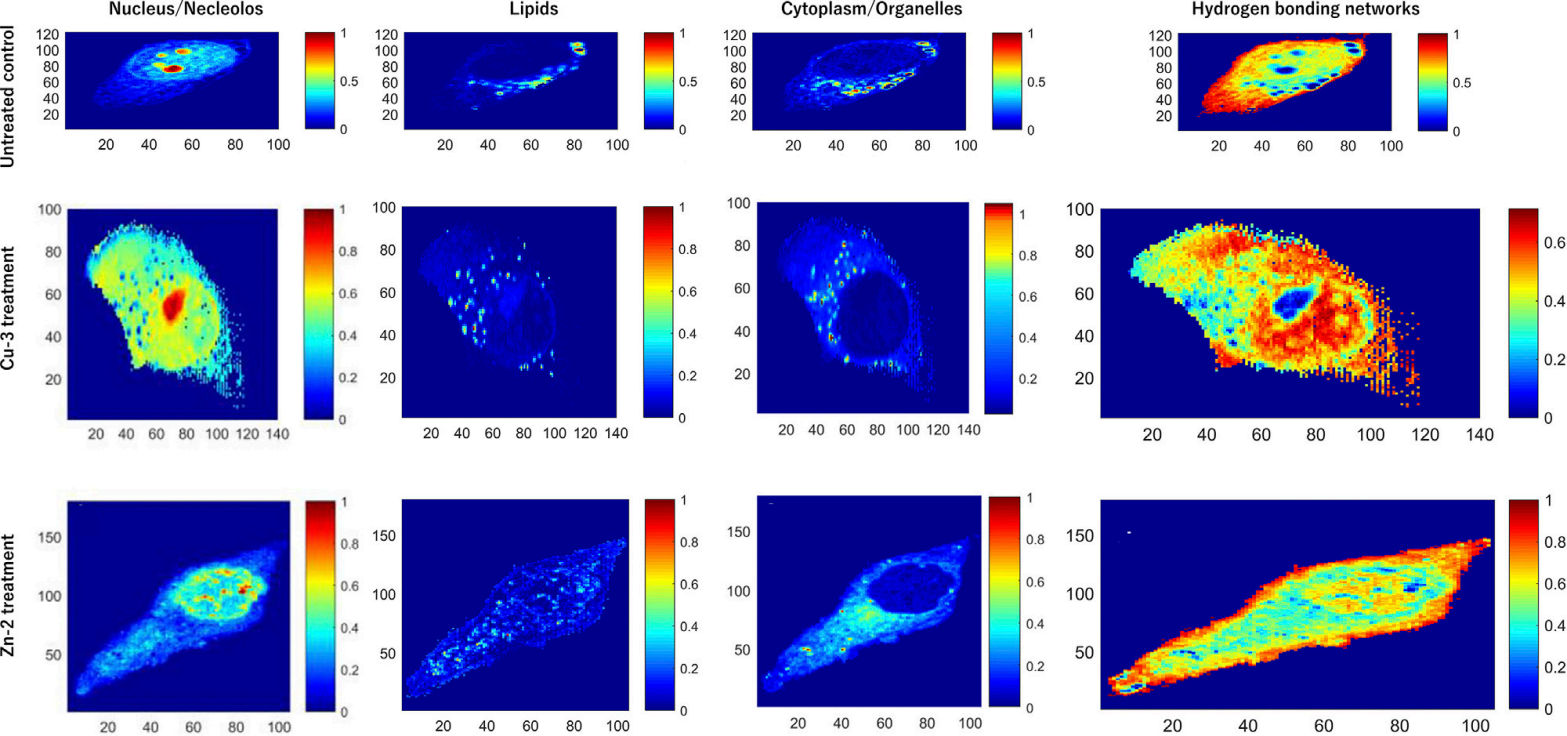
Raman images of untreated HeLa cells (control) and cells treated with Cu-3 and Zn-2. Intracellular components are assigned based on multivariate curve resolution with alternate least squares method (MCR-ALS). Four profiles are assigned in each case: nucleus, lipids, cytoplasm, and hydrogen bonding networks. Adapted from Mamián-López et al., 2021. Reproduced with permission from Elsevier.

More recently, the cellular damage caused by compounds Cu-3 and Zn-2 in living HeLa cells were monitored by confocal Raman microscopy coupled to multivariate curve resolution with alternate least square method (MCR-ALS) (Mamián-López et al., 2021). With this approach, it was possible to extract the characteristic spectral pattern of each intracellular component and assess their distribution in the untreated and treated cells. Four principal components (PC) could explain at least 90% of the Raman data variance. A non-treated control, corresponding to untreated HeLa cells was analyzed, and it was possible to make spectra assignments corresponding to the nucleus, lipids, cytoplasm, and hydrogen bonding (Figure 10). Upon treatment with Cu-3 (16 µM, 24 h), the nucleus and nucleolus content outflowed to the cytoplasm, followed by the releasing of cytochrome c, a remarkable effect that was not observed upon treatment with Zn-2 (100 µM, 24 h). Cu-2 caused alterations in the cytochrome c response (as part of the lipids components), which is not observed for Zn-2. Finally, the hydrogen bonding network between water molecules and the biomolecules present in the different cellular compartments was probed. For Cu-3-treated cells, an expansive hydrogen bonding network spanning throughout almost the whole cell can be observed. In contrast, this is not observed for Zn-2-treated cells, where the cellular integrity is still holding upon treatment with this compound. A much larger extent of induced damage was verified upon treatment with Cu-3. These data suggest that depending on the metal, different mechanisms can be working. Taken together, this approach corroborates the apoptotic mechanism of cell death by Cu-3 monitored by other techniques, and previously reported (Filomeni et al., 2007).

##### Proteins as Potential Biological Targets

Besides DNA and mitochondria, it was also demonstrated that the isatin-based coordination compounds have other important targets. Some of them were identified and discussed below. In addition, it was found that these compounds act as multifunctional agents, and their intracellular speciation can be vital to understand their modes of action.

###### Human Topoisomerase IB

Human topoisomerases, proteins involved in DNA replication, transcription, recombination, and chromatin remodeling, by introducing temporary single- or double-strand breaks in the DNA, are also targets in diverse anticancer investigations (Champoux, 2001). Topoisomerase IB (topo IB) is the target of a vast number of drugs that depending on their action are divided as poisons and inhibitors. Topotecan and irinotecan, camptothecin analogs and topoisomerase IB-targeting molecules have entered the clinical practice for the treatment of many human cancers (Pommier, 2006; Thomas and Pommier, 2019).

**FIGURE 11:**
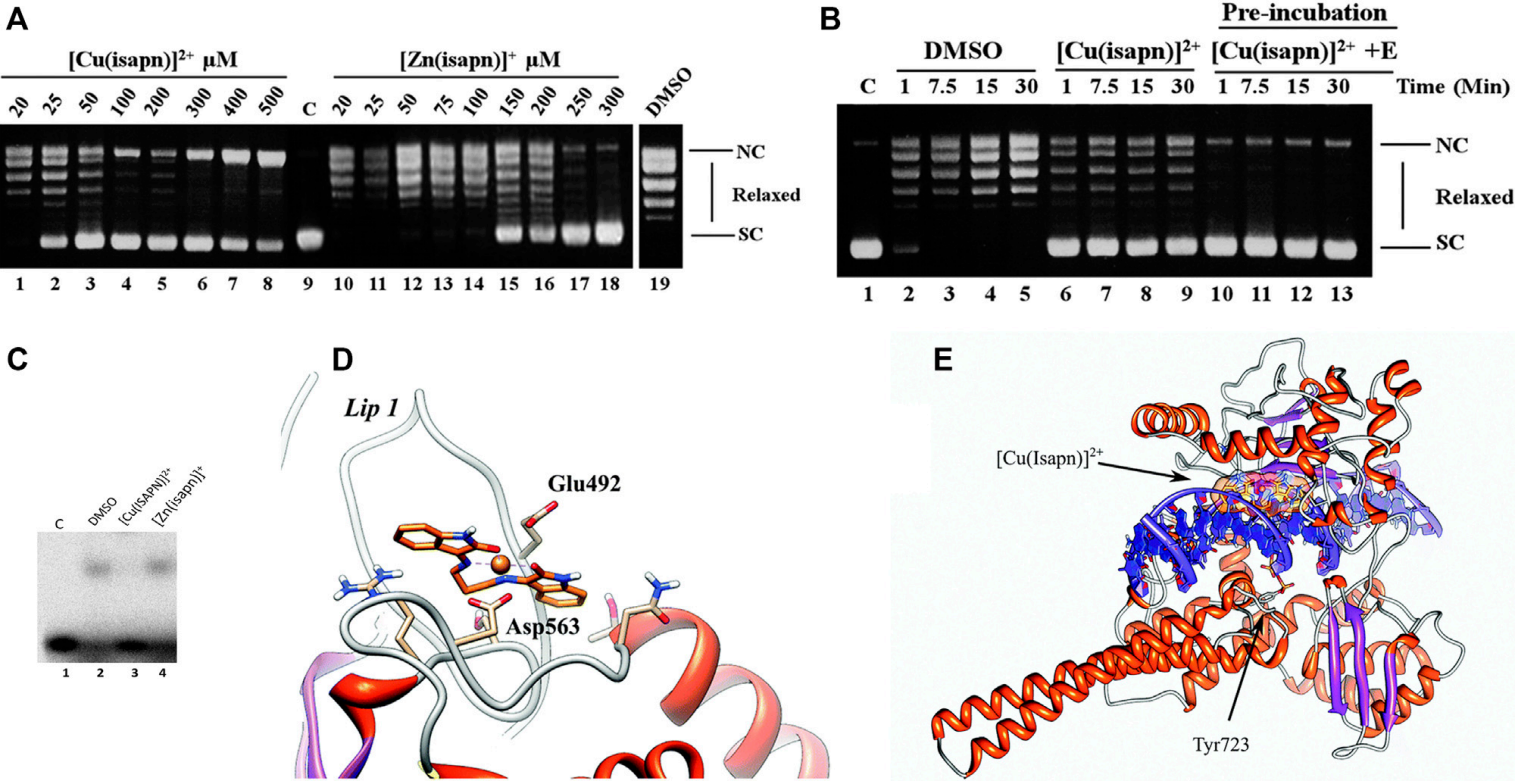
**(A)** Relaxation of negative supercoiled plasmid DNA by topoisomerase IB in the presence of increasing concentration of Cu-1 (lanes 1–8) and its Zn(II) analog (lanes 10–18). The reaction products were resolved in an agarose gel and visualized with ethidium bromide. Lane 9, no protein added. Lane 19, control reaction with DNA and DMSO without Cu-1 and its Zn(II) analog. NC, nicked circular plasmid DNA. SC, supercoiled plasmid DNA. **(B)** Relaxation of negative supercoiled plasmid DNA in a time course experiment with DMSO (lanes 2–5), in the presence of 25 μM Cu-1 (lanes 6–9), after pre-incubation of 25 μM Cu-1 with the enzyme for 5 minutes at 37°C (lanes 10–13). Lane 1, no protein added. **(C)** Electrophoretic mobility shift assay of the [γ^−32^P] radiolabeled substrate CL25/CP25 alone (lane 1), in the presence of inactive mutant Y723F enzyme (lane 2), in the presence of the mutant and 50 μM Cu-1 (lane 3), in the presence of the mutant and 300 μM of the Zn(II) analog to Cu-1 (lane 4). **(D)** Representative structure of the most populated cluster for the Cu-1-topoisomerase IB docking. The Glu492 and Asp563 residues located on lip1 and coordinating the Cu-1 are labeled and represented as sticks. **(E)** Representative structure of the most populated cluster for the docking of Cu-1 on the binary (topoisomerase I + DNA) covalent complex docking. Cu-1 is labeled and represented as orange sticks. The protein and the DNA backbone are represented as ribbons. Catalytic Tyr723 of topoisomerase I is labeled and represented as sticks. Reproduced from Katkar et al., 2014 with permission from The Royal Society of Chemistry.

The effect of compound Cu-1 and its Zn(II) analog on Topo IB was verified by plasmid relaxation assays, indicating that both compounds inhibit the relaxation activity of this enzyme (Figure 11A). This effect is enhanced to the point of total cleavage inhibition when the enzyme is pre-incubated with the metal complex before substrate addition (Figure 11B). At 50 µM, Cu-1 fully inhibits the relaxation of topoisomerase IB, while the analogous zinc compound shows a similar effect, although at a much higher concentration (300 µM). Such differences in inhibition potencies are most likely related to the geometries of the compounds, as verified in docking simulations. Cu-1 has an almost square planar geometry, based on EPR data, which allows for easy access to residues Glu492 and Asp463 (Figure 11D) forming a stable octahedral adduct with the enzyme. This binding hinders the interaction of the enzyme to DNA (Figure 11C, docking simulation shown in Figure 11E), in the case of Cu-1, but not for the Zn(II) analog, that adopts a distorted tetrahedral coordination.

Additional studies with Cu-3 and Zn-2 (isaepy-containing compounds) pointed to different mechanisms of inhibition in the relaxation step catalyzed by topo IB (Castelli et al., 2018). Cu-3 was found to be a more potent relaxation inhibitor than Zn-2. Pre-incubation experiments demonstrated an increased inhibitory activity only for Cu-3, indicating a reversible binding of this compound to the enzyme. The same trend was observed when studying the cleavage reaction. Finally, both compounds were not inhibitors of religation step. The compound Cu-3 also inhibited the formation of a non-covalent topoisomerase-DNA adduct, contrasting to what was observed for Zn-2.

In further studies, a vanadyl analog was investigated as potential inhibitor of topo IB (compound V-1, Figure 8) (Dario et al., 2019). V-1 was a potent cytotoxic agent against the human colon HCT-116 cell line (IC_50_ = 7.98 ± 1.30 µM) and human breast MDA-MB-231 cell line (IC_50_ = 6.58 ± 7.68 µM), while being non-toxic (IC_50_ > 100 µM) against the non-tumorigenic fibroblast P4. When targeting Topo IB, V-1 was shown to preferentially bind to the enzyme (vs. DNA), inhibiting the cleavage step, but not the religation step, similarly to what was observed with the other metal compounds, Cu-3 and Zn-2. CT-DNA melting temperature (T_m_) assays further confirmed that V-1 does not interact significantly with DNA. The Topo 1 inhibitory activity of V-1 appears between the highly inhibitory Cu-1 and the less inhibitory Zn-1 analogue (Dario et al., 2019). Differences in the inhibitory potencies observed for these compounds toward Topo IB are related to their distribution among the preferential binding sites in the enzyme, as indicated by computational studies (molecular docking). Three main protein regions were identified for the interaction of these compounds in the core subdomain III of the Topo IB: the 1) “external region;” 2) lips and 3) center channel. The most active compound interacts predominantly at this “external region”, next to the catalytic Tyr723, causing more efficient distortion of the protein environment and consequently reduction of the protein catalytic activity.

###### Kinases as Targets

The activity of oxindolimine-metal complexes was also monitored *in vitro* over cyclin-dependent kinase (CDK1), responsible for the phosphorylation of other proteins in cells (Miguel et al., 2015). Results using histone H1 as substrate indicated a concentration dependence. In general copper(II) compounds have a more prominent inhibition of CDK1 kinase activity than the zinc(II) analogs or the free ligand. At 5 µM Zn-2, [Zn(isaepy)]_2_, caused 10 % inhibition of the kinase activity like that of the Zn-free ligand, whereas at 50 µM > 90 % inhibition was achieved. On the other hand, at 5 µM Cu-3, [Cu(isaepy)], caused 65 % inhibition of CDK1 activity, whereas at 50 µM once again >90% inhibition was observed. Another series of compounds, bearing the isapn ligand, was also studied. At 50 µM, the free isapn inhibited CDK1 activity by 20%, while at 50 µM Zn-1, [Zn(isapn)], led to a 30 % inhibition. Cu-1, at the same concentration of 50 µM led to 80% inhibition. These differences can be assigned to a combination of geometric factors [tetragonal /square planar coordination sphere of Cu(II) vs. mainly tetrahedral for Zn(II)], and a higher cellular uptake of the isaepy-containing compounds when compared to the isapn-containing ones. This study points CDK-1 (and kinases as a family) probably as a crucial biological target of these compounds.

Complementary, to determine whether the activity of the compounds is specific only to the phosphorylation reaction, it was also investigated the effect of the compounds on the dephosphorylation activity of alkaline phosphatase on the histone H1. However, the whole series of compounds studied here had little to no effect in the dephosphorylation reaction (Miguel et al., 2015).

###### Galectin-3

The cytotoxic effect of Cu-2 against melanoma cells was evaluated using a model of vertical growth melanoma (TM1), in which galectin-3 (GAL3) expression is lost during tumor progression (Borges et al., 2013). The results demonstrated that *de novo* galectin-3 expression impairs the cellular antioxidant system and renders TM1G3 cells more susceptible than GAL3-null TM1MNG3 cells to Cu-2 treatment. This compound, in contrast with the redox inactive Zn-2, leads to an increased intracellular ROS accumulation, increased carbonyl stress, increased mitochondrial depolarization, decreased cell adhesion, increased p38 activation, and induced apoptosis in TM1G3 cells. Cu-2 was found to target mitochondria, as previously observed with neuroblastomas, and generate intracellular hydrogen peroxide-derived species, favored by the presence of galectin-3 in these organelles. Apoptosis here is also related to p38 MAPK activation.

#### Heterobimetallic Oxindolimine Compounds of Cu(II) and Pt(II)

**FIGURE 12:**
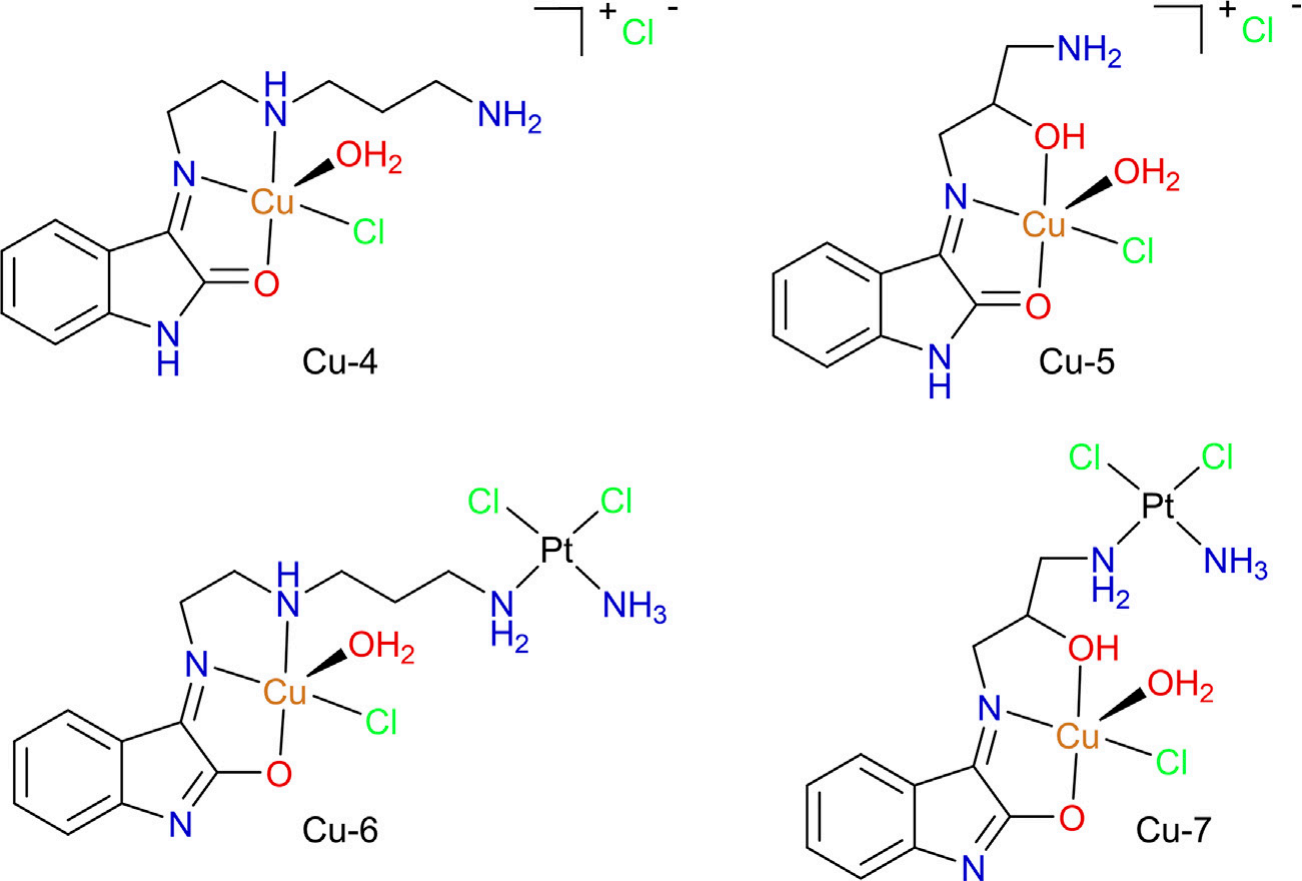
Cu(II)/Pt(II) heterobimetallic compounds (Cu-6 and Cu-7) with isatin-derivatives as ligands, isolated as enolate forms, and corresponding copper-only precursors (Cu-4 and Cu-5), obtained as keto forms.

The mononuclear copper(II) compounds Cu-4 and Cu-5 and the analogous heterobimetallic Cu-6 and Cu-7, shown in Figure 12, were designed and prepared to study the possible advantages of combining a “cisplatin-like” DNA-cross linking motif with the copper(II)-oxindolimine motif developed in our lab (Aranda et al., 2016). Similar investigations of dinuclear metal compounds involving imine or amine ligands (but not the oxindole moiety) have been reported in the literature (De Hoog et al., 2007; Zhu et al., 2009).

EPR spectroscopy indicated a square pyramidal geometry for Cu-4 and Cu-5, while a more tetragonal geometry was observed around the copper(II) centers in the heterobimetallic analogues. CD spectroscopy indicated that Cu-6 and Cu-7 indeed lead to more prominent changes in the secondary structure of calf thymus DNA (CT-DNA) in comparison to Cu-4 and Cu-5.

In terms of cytotoxicity, all compounds showed remarkable activity against the highly invasive B16F10 melanoma cell line. Combination with platinum(II) led to lower IC_50_ values in all cases, with Cu-6 being the most potent cytotoxic agent in the series with IC_50_ = 0.63 ± 0.25 µM, compared to IC_50_ of ∼2 µM, for Cu-4 and Cu-5. A similar behavior was observed against the human sarcoma MS-SA and MES-SA/Dox5 (resistant) cell lines. The overall higher cytotoxicity observed for Cu-6 can be related to the O, N, N chelating motif but also with the higher lipophilicity introduced by the longer alkylamine chain, when compared to Cu-7.

This series of compounds was further evaluated in terms of DNA binding, as well as kinase and phosphatase inhibition properties (Aranda et al., 2020). Fluorescence assays showed that mononuclear compounds Cu-4 and Cu-5 have no effect on ethidium displacement from calf thymus DNA (CT-DNA). On the other hand, the Pt(II) conjugates Cu-6, and Cu-7 are capable of competing for ethidium binding sites. The effect was most prominent for Cu-6, comparable to cisplatin. The oxidative cleavage of the plasmid pBluescript II in the presence of H_2_O_2_, at 6 µM and 60 min incubation, shows significant differences between the two mononuclear copper compounds. Cu-4 leads to a double cleavage, with the open circular form prominent (with much supercoiled plasmid still present). On the other hand, Cu-5 leads primarily to the nicked form, but with a complete cleavage of the supercoiled form. Among the heterobimetallic compounds, a combination of effects typical to copper(II) and platinum(II) compounds could be observed. Under the same conditions, Cu-6 incubation stabilizes the supercoiled form of the plasmid, while for Cu-7 a single oxidative cleavage is observed (owing to copper), but accompanied by a significant smear of the nicked DNA band (which is typical of platinum(II) binding). When targeting histone H1 phosphorylation by CDK1, the mononuclear compounds Cu-4 and Cu-5 were the most active in the series with significant inhibition observed at 50 µM. The heterobimetallic compounds, on the other hand, had little to no inhibitory effect. Regarding the dephosphorylation step, evaluated using the alkaline phosphate assay, all the complexes had minor effects on the alkaline phosphatase activity (Aranda et al., 2020). These results are indicative that cyclin-dependent kinases are crucial targets for that class of compounds, in contrast to alkaline phosphatase proteins.

### Metallocenes Containing Isatin-Related Motifs

Besides traditional coordination compounds, organometallic examples containing isatin-derivatives were also investigated as anticancer agents.

**FIGURE 13:**
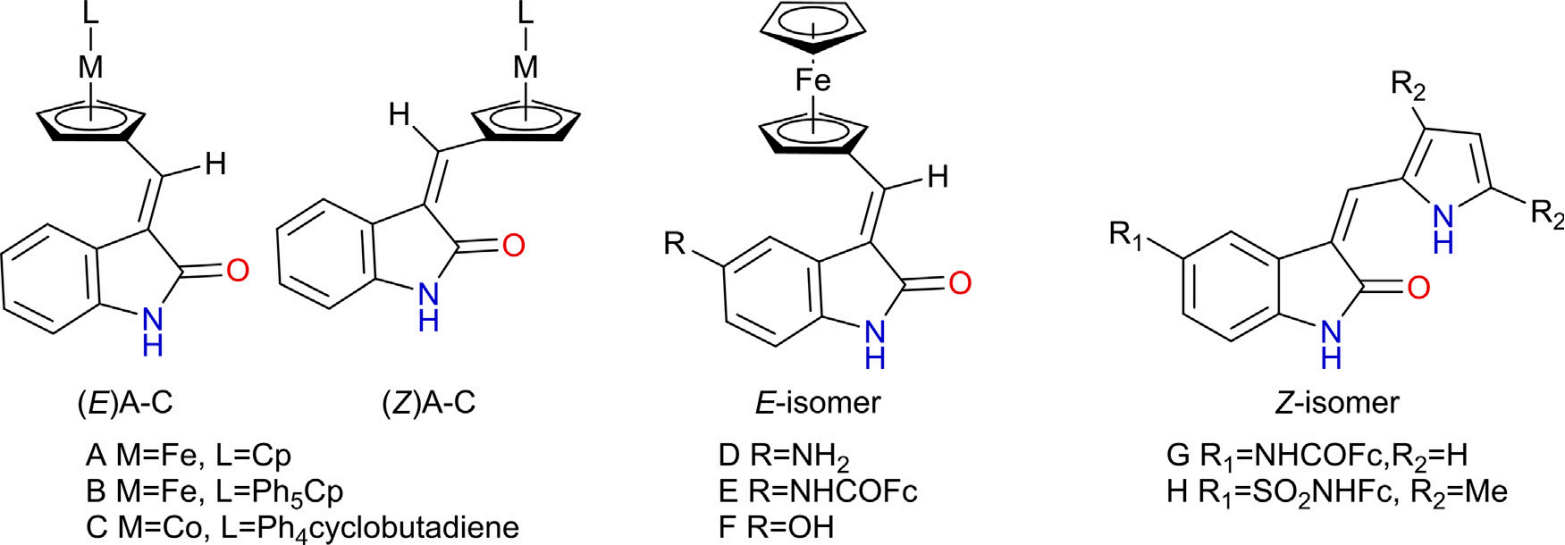
Some 2-oxindole-functionalized metallocene compounds developed by Spencer et al., 2011a; Spencer et al., 2011b, as kinase inhibitors.

A series of metallocene compounds functionalized with 2-oxindole [(E) A-C and (*Z*) A-C, Figure 13], inspired by the structures of Sunitinib and Semaxanib, were reported in the literature (Spencer et al., 2011a; Spencer et al., 2011b). The oxindole group was introduced as a kinase inhibiting motif aiming at improving the anticancer potency of the compounds. These compounds were obtained as a mixture of *E*- and *Z*-isomers, that were readily separated by chromatography. The antiproliferative activity of each isomer was evaluated against K592 leukemia cells. The metal center and the identity of ligand were found to be more important for the cytotoxicity observed experimentally than the *E*,*Z*-isomerism. In this series, the iron compounds bearing the cyclopentadienyl (Cp) ligand were the most cytotoxic (E-A and *Z*-A).

When targeting kinases, the inhibitory activity of the compounds was first evaluated against kinases p21-activated kinase-1 (PAK1), which contains an ATP binding domain and can accommodate octahedral metal complexes. At 10-30 μM, none of the compounds exhibited appreciable inhibitor activity against PAK1. On the other hand, the compounds E-A and *Z*-A were active against DYRK3 and DYRK4 (Spencer et al., 2011a). In a larger screening against a panel of 50 kinases (Spencer et al., 2011a; Spencer et al., 2011b), E-A and *Z*-A had almost no cross-inhibition. The authors highlight as notable inhibition of DYRK4 by both E-A and *Z*-A; while *Z*-A inhibits DYRK3 with an IC_50_ value of 390 nM, as opposed to the lack of appreciable inhibition observed for E-A (see Supplementary Table S1).

This series was further explored by the introduction of different substituents, with crystal structures solved for most of the compounds (Amin et al., 2013). These compounds were screened against VEGFR-2 and four isoforms of kinase DYRK, and the results obtained by the authors are summarized in Supplementary Table S1, in comparison to Sunitinib. Complexes E-E and *Z*-E, containing two ferrocenes, were poor VEGFR-2 inhibitors but potent inhibitors of DYRK isoforms 3 and 4. *Z*-G, on the other hand, displays inhibition of VEGFR2 in nM range, and single-digit µmolar inhibition of DYRK isoforms 2−4.

## Carriers for Modified Delivery of Isatin-Derivatives

**FIGURE 14:**
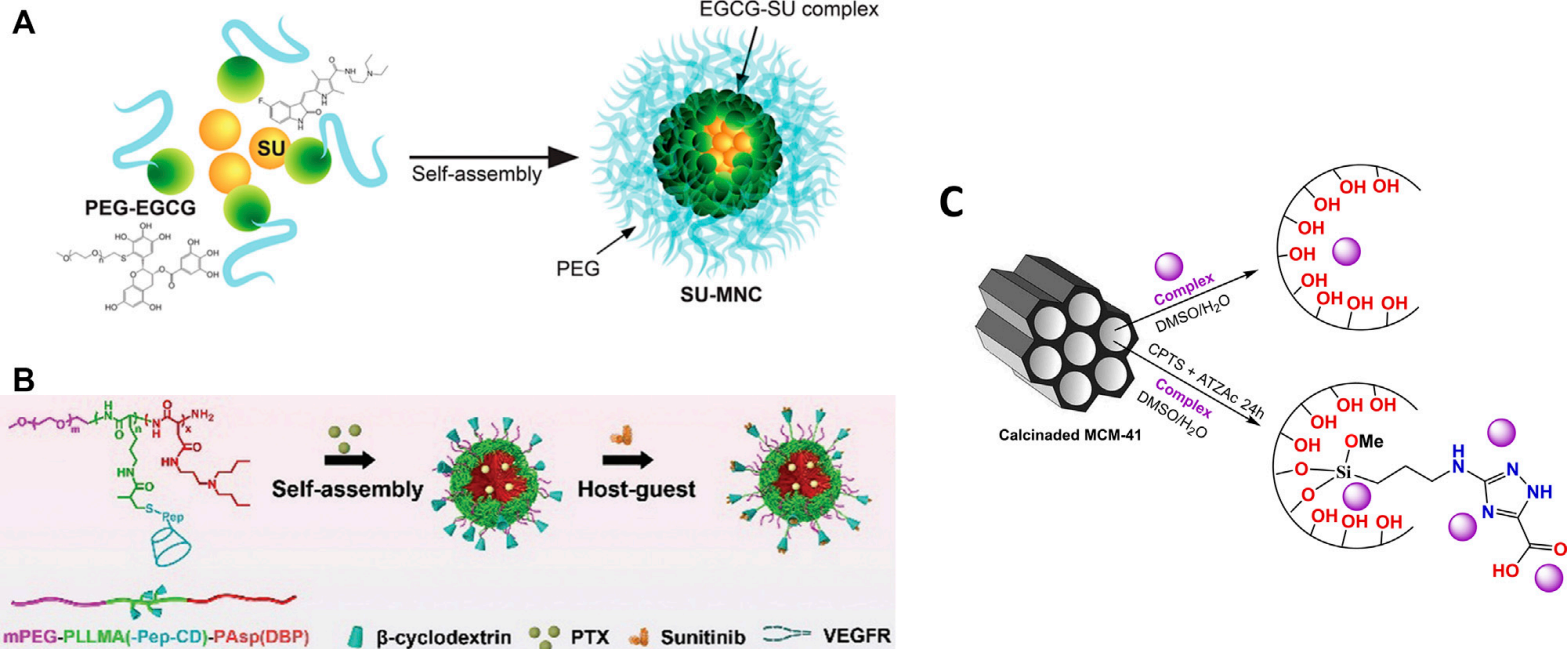
**(A)** Schematic diagram of SU-MNC self-assembled from SU and PEG-EGCG. Reprinted with permission from Yongvongsoontorn et al., 2019 (Copyright, American Chemical Society). **(B)** PTX and Sunitinib co-loaded micelle (PTX-Sunit-psMC). Reproduced from He et al., 2019 with permission from The Royal Society of Chemistry; **(C)** Scheme of immobilization of isatin-based complexes into unmodified and modified silica carrier (MCM-41), based on Vieira et al., 2019.

Several drug delivery systems have been developed as an alternative to improve the performance of compounds with recognized antitumor activity. This improvement in performance has been designed by the possibility of increasing efficiency, greater stability through the formation of hybrid compounds or greater selectivity. As a representative example of a clinically approved isatin-related compound, systems reported in the literature for sunitinib (Figures 14A,B), are presented in Section *Sunitinib Delivery Systems*.

However, only few works in the literature reported the loading of isatin-based metal complexes for antitumoral applications (Figure 14C). This topic is further explored in Section *Oxindolimine-Metal Complexes Supported in Functionalized Silica*.

### Sunitinib Delivery Systems

Loading organic drugs into several nanometric drug delivery systems has been reported as an interesting alternative for increasing the efficiency and minimizing the toxicity of a drug, prolonging the exposure time to lower doses, increasing selectivity and decreasing side effects in patients (Patra et al., 2018; Singh et al., 2019). To paint a better picture on the effect of drug delivery systems on oxindole-containing compounds, we explore here some examples of delivery systems for the clinically approved drug Sunitinib as a case study.

One of the main problems that decrease the efficiency and increase the toxicity in drug delivery in the treatment of cancer is the low percentage of drug loaded, which usually leads to the need to apply a larger dose of the drug. A counterpoint alternative may arise when the carrier itself presents cytotoxicity and there is a synergism between drug and carrier, leading to an enhancement in activity. Sunitinib (SU) a therapeutic drug already approved for the treatment of cancer, was encapsulated in micelles containing epigallocatechin-3-O-gallate (EGCG) (see Figure 14A), a component of green tea with antitumor properties. The complex formed (SU-MNC) exhibited a better activity and reduced toxicity in comparison to the conventional treatment with Sunitinib (Yongvongsoontorn et al., 2019). High antiproliferative effects with similar concentrations of SU and SU-MNC were observed toward human umbilical vein endothelial cells (HUVECs) in normal and VEGF-induced growth conditions, a determining factor in angiogenesis and tumor growth. In addition, SU-MNC showed a significant reduction in toxicity towards human renal proximal tubule epithelial cells (HRPTECs) compared to SU. *In vivo* studies of SU, SU-MNC and SU + empty MNC (physical mixture) in tumor-xenografted mice indicated more significant effects at high doses of SU and SU-MNC, demonstrating a synergism between the drug and the carrier. Further, SU-MNC showed a most significant proliferation reduction, increased apoptosis, and lower tumor microvascular density values in tumor cells.

The proliferation of human umbilical vein endothelial cells (HUVECs) in normal and VEGF-induced growth conditions was verified in the presence of SU and SU-MNC. Both showed higher antiproliferative effects with similar concentrations in VEGF-induced conditions, what is expected when these pathways are active, which is a determining factor in the angiogenesis and tumor growth. Next step, the effect of SU, SU-MNC and SU + empty MNC (physical mixture) in tumor-xenografted mice was investigated. The treatment showed that the effect is more significant at high doses of SU and SU-MNC demonstrating the synergism between the drug and the carrier. In addition, SU-MNC shows the most significant reduction in tumor cell proliferation, increased apoptosis in tumor cells and lower tumor microvascular density values.

Another positive aspect observed, was the selectivity of the SU-MNC, which presents a higher concentration in tumor compared to SU, and lower concentrations in other organs such as skin, muscle, lung, and stomach in ACHN / A498-xenografted mice until 8 h post-injection. In comparison, another conventional nanocarrier (SU-PM), SU-MNC showed an increase in tumor growth inhibition and Sunitinib concentrations in tumors in athymic nude mice.

In addition, a new tumor microenvironment (TME) responsive polymeric micelle loaded with SU was developed (He et al., 2019) to obtain a programmed delivery specific to the site of angiostatin (see Figure 14B). In this study, polymeric micelles conjugated to β-cyclodextrin molecules can release sunitinib to target endothelial cells in the tumor extracellular matrix, and paclitaxel (PTX) into cancer cells. Therefore, the efficient anti-angiogenesis effect of SU and PTX to tumor cells was obtained due to a great synergism between both drugs, resulting in an extremely effective tumor treatment. According to the authors, the release of the anti-angiogenesis SU occurs because the matrix metalloproteinase 2 (MMP-2) overexpressed cleaves the peptide/β-CD linker, causing an effective vascular shutdown (He et al., 2019).

### Oxindolimine-Metal Complexes Supported in Functionalized Silica

In our studies of antitumor properties of oxindolimines, all different strategies considered in item 3 were tested, and permitted some improvements in the reactivity of such compounds. To the best of our knowledge, our group is the first to report the preparation of two oxindolimine-metal complexes Cu-1 and Zn-1, in keto-enol form, and its loading into two distinct biocompatible materials, such as unmodified and modified silica MCM-41 for delivery of the active compounds, as shown in Figure 14C (Vieira et al., 2019).

**FIGURE 15:**
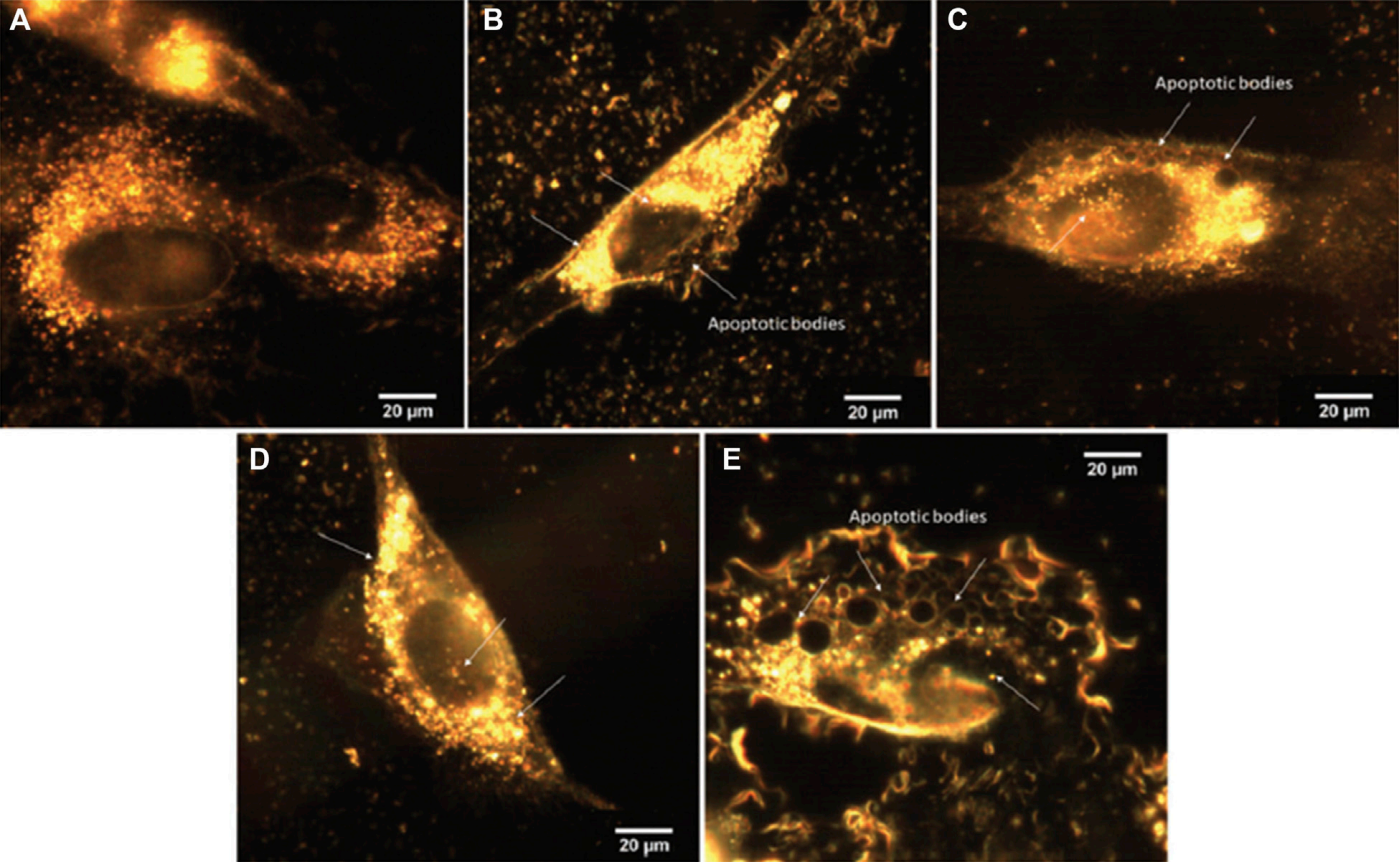
Optical images of SK-MEL-147 cells (registered with a CytoViva instrument), incubated for 24 h with different materials. **(A)** Control, **(B)** Cu-1@MCM, **(C)** Zn-1@MCM, **(D)** Cu-1@MCM-atzac, and **(E)** Zn-1@MCM-atzac nanoparticles. Arrows indicate the localization of metal-loaded NPs (bright points) or apoptotic bodies (dark circles). Reproduced from Vieira et al., 2019 with permission from The Royal Society of Chemistry.

The free and loaded materials were tested against two different melanoma cells lines, SK-MEL-147 (wild type) and SK-MEL-05 (B-Raf mutated gene expression), in addition to the cervical HeLa cells, in comparison to non-tumor cells line, P4 Fibroblast. The free complexes did not present significant activity up to 100 µM, except for Cu-1 against HeLa cells, with IC_50_ = 80 ± 6 µM, after 24 or 48 h treatment (Vieira et al., 2019). Furthermore, the antiproliferative assays for loaded materials, Cu-1@MCM, Zn-1@MCM, Cu-1@MCM-atzac, Zn-1@MCM-atzac, showed a high selectivity toward SK-MEL-147 cells for both unmodified and modified MCM-41. The selectivity index indicated at least twice more cytotoxicity to the tumor cells SKMEL-147, at both incubation times. Taking into account the percentage (mass/mass) of loaded complexes into the matrices and 100 % release, the IC_50_ values were determined, being much lower compared to those of the free complexes, (70.8 ± 2.4 µM) and (9.3 ± 0.3 µM) for Cu-1 inserted into unmodified and modified MCM-41, respectively. Further, the zinc analog shows to be much more cytotoxic, (55.8 ± 5.9 µM) and (4.4 ± 1.2 µM) for Zn-1 loaded into both matrices. Complementing these results, optical images in CytoViva assays with the SK-MEL-147 cells treated with loaded materials (24 h incubation) showed nanoparticles both in the cytoplasm and in the nucleus, as well as the appearance of apoptotic bodies in cells, as shown in Figure 15 (Vieira et al., 2019).

DNA damage was monitored by the alkaline comet assay, using the untreated cells as control, in addition to those treated with aqueous copper(II) salt (Vieira et al., 2019). These controls showed no or tiny DNA damage, while the pure matrix (MCM-41) causes twice as much DNA damage in comparison. These data show an increase in DNA damage in the presence of matrix-complex materials compared to the free matrix, indicating that the matrix is probably stimulating the transport of the complex into the cell, since the free complexes have no measurable activity against SK-MEL-147. Therefore, a synergism occurs between the matrices and the metal complexes, increasing the cytotoxicity of these complexes.

## Conclusion and Perspectives

Oxindoles, and particularly isatin, are promising scaffold for pharmacological and medicinal compounds, according to the vast range of different biological properties this kind of compounds exhibit. Beyond the diversity of their activities, it is remarkable the relationship observed between their structural features, with possibilities of straightforward structural changes, and the improvement achieved in their reactivities in more recent investigations. Introduction of imine, hydrazone, thiosemicarbazone moieties seems to ameliorate their antiproliferative properties, through the better recognition of such compounds by crucial biomolecules (DNA and proteins). Particularly, the reactivity of non-metallated, as well as of metal-containing derivatives, were explained, or previewed in many simulation studies (DFT studies, docking, molecular dynamics), supporting experimental data.

Metal complex formation is a more noticeable strategy in the direction of reactivity enhancement, including traditional coordination compounds as well as organometallics. Different metal complexes containing the oxindole moiety were isolated as keto- and enolate-species, allowing to verify differences in its biological activities, although at physiological pH an equilibrium between both forms is usually observed. Metal ions introduce charge, provide a more rigid geometry to the ligands, and additional binding sites to the biomolecules, facilitating interactions.

Finally, the accomplishment of adequate carrier systems for an effective modified delivery of such compounds can be of great efficacy, in a synergistic process where the matrices act as adjuvants, facilitating the metal uptake by the cells and allowing an efficient modulation of their release inside the cell.

Although a huge number of studies have been published on the subject, not all the investigations explore the molecular bases or modes of action, aiming to explain the antitumor activity of such compounds. Studies focusing DNA damage are recurrent both with non-metalated and metalated isatin-derivative compounds. Among the proteins inhibited by these compounds, several types of kinases appear as relevant and preferential targets. Additional targeted proteins include topoisomerases I and II, and phosphatases.

In our studies of antitumor properties of oxindolimines, all these strategies were tested, and permitted a rational development of new compounds with diverse mechanisms of action, as well as some improvement in their reactivity. Also, diverse targets were investigated (DNA, mitochondria, CDKs, topoisomerase IB, alkaline phosphatase), inducing cell death by apoptosis. In our investigations, as well as in similar results with different ligands reported in the literature, the metal complex was more active than the corresponding free ligand, attesting that metalation is an efficient strategy.

Despite the continuous efforts already described in elucidating their chemical and biological properties, further rational investigations on isatin-derivatives in its different forms, metal-free, corresponding metal complexes or supported species, can lead to more efficient drugs presenting lower toxicity, and to better elucidation of their multiple mechanisms of action.

## Author Contributions

AF conceived the structure of the manuscript, the literature search criteria, completed and revised the manuscript. RP focused on the metal complexes and metallocenes, and revised the manuscript; EV discussed inserted or supported materials; DS was mainly responsible for the discussion of approved oxindole-based drugs; CW performed most of bibliographic search, and contributed to the analysis of non-metallated oxindoles. All authors contributed substantially to the article and approved the submitted version.

## Funding

All the authors are grateful to São Paulo State Research Foundation (FAPESP, grant 2013/07937-8) for financial support. EV and RP are also grateful to FAPESP for post-doc fellowships (FAPESP 2016/16735-8 and 2018/21537-6, respectively). DS and CW thank CAPES (Coordenação de Aperfeiçoamento de Pessoal de Nível Superior, Finance code 01) for PhD fellowships.

## Conflict of Interest

The authors declare that the research was conducted in the absence of any commercial or financial relationships that could be construed as a potential conflict of interest.

The University of São Paulo has a Brazilian patent (BR PI 0600985-9, conceded on March 24th, 2020–INPI Brazil) based on the oxindolimine-metal complexes with antitumor activity, designed and prepared at AF laboratory, at Institute of Chemistry-USP.

## References

[B1] Abdel-Aziz H. A. Aboul-Fadl T. Al-Obaid A. R. Ghazzali M. Al-Dhfyan A. Contini A. (2012). Design, synthesis and pharmacophoric model building of novel substituted nicotinic acid hydrazones with potential antiproliferative activity. Arch. Pharm. Res. 35, 1543–1552. 10.1007/s12272-012-0904-2 23054710

[B2] Alafeefy A. M. Ashour A. E. Prasad O. Sinha L. Pathak S. Alasmari F. A. (2015). Development of certain novel N-(2-(2-(2-oxoindolin-3-ylidene)hydrazinecarbonyl)phenyl)-benzamides and 3-(2-oxoindolin-3-ylideneamino)-2-substituted quinazolin-4(3H)-ones as CFM-1 analogs: design, synthesis, QSAR analysis and anticancer activity. Eur. J. Med. Chem. 92, 191–201. 10.1016/j.ejmech.2014.12.048 25555142

[B3] Ali M. A. Mirza A. H. Bakar H. J. H. A. Bernhardt P. B. (2011). Preparation and structural characterization of nickel(II), cobalt(II), zinc(II) and tin(IV) complexes of the isatin Schiff bases of S-methyl and S-benzyldithiocarbazates. Polyhedron 30, 556–564. 10.1016/j.poly.2010.11.016

[B4] Ali A. Q. Eltayeb N. E. Teoh S. G. Salhin A. Fun H. K. (2012). Bis[2-(2-oxoindolin-3-yl-idene)-N-phenylhydrazinecarbothio-amidato-κ(3)O,N(2),S]nickel(II) dimethyl-formamide monosolvate. Acta Crystallogr Sect E Struct Rep Online 68, m538–m539. 10.1107/S1600536812012834 PMC3344298 22590064

[B5] Amin J. Chuckowree I. S. Wang M. Tizzard G. J. Coles S. J. Spencer J. (2013). Synthesis of oxindole-based bioorganometallic kinase inhibitors incorporating one or more ferrocene groups. Organometallics 32, 5818–5825. 10.1021/om400359m

[B6] Aneja B. Khan N. S. Khan P. Queen A. Hussain A. Rehman M. T. (2019). Design and development of Isatin-triazole hydrazones as potential inhibitors of microtubule affinity-regulating kinase 4 for the therapeutic management of cell proliferation and metastasis. Eur. J. Med. Chem. 163, 840–852. 10.1016/j.ejmech.2018.12.026 30579124

[B7] Ansari J. Fatima A. Fernando K. Collins S. James N. D. Porfiri E. (2010). Sunitinib in patients with metastatic renal cell carcinoma: birmingham experience. Oncol. Rep. 24, 507–10. 10.3892/or_00000886 20596640

[B8] Aranda E. E. da Luz J. S. Oliveira C. C. Petersen P. A. D. Petrilli H. M. da Costa Ferreira A. M. (2020). Heterobinuclear copper(II)-platinum(II) complexes with oxindolimine ligands: Interactions with DNA, and inhibition of kinase and alkaline phosphatase proteins. J. Inorg. Biochem. 203, 110863 10.1016/j.jinorgbio.2019.110863 31683126

[B9] Aranda E. E. Matias T. A. Araki K. Vieira A. P. de Mattos E. A. Colepicolo P. (2016). Design, syntheses, characterization, and cytotoxicity studies of novel heterobinuclear oxindolimine copper(II)-platinum(II) complexes. J. Inorg. Biochem. 165, 108–118. 10.1016/j.jinorgbio.2016.08.001 27503192

[B10] Azizian J. Mohammadi M. K. Firuzi O. Razzaghi-Asl N. Miri R. (2012). Synthesis, biological activity and docking study of some new isatin Schiff base derivatives. Med. Chem. Res. 21, 3730–3740. 10.1007/s00044-011-9896-6

[B11] Balachandran C. Haribabu J. Jeyalakshmi K. Bhuvanesh N. S. P. Karvembu R. Emi N. (2018). Nickel(II) bis(isatin thiosemicarbazone) complexes induced apoptosis through mitochondrial signaling pathway and G0/G1 cell cycle arrest in IM-9 cells. J. Inorg. Biochem. 182, 208–221. 10.1016/j.jinorgbio.2018.02.014 29510336

[B12] Barra F. Laganà A. S. Ghezzi F. Casarin J. Ferrero S (2019). Nintedanib for advanced epithelial ovarian cancer: a change of perspective? summary of evidence from a systematic review. Gynecol. Obstet. Invest. 84, 107–117. 10.1159/000493361 30304728

[B13] Bayly M. J. Duretto M. F. Holmes G. D. Forster P. I. Cantrill D. J. Ladiges P. Y. (2015). Transfer of the New Caledonian genus Boronella to Boronia (Rutaceae) based on analyses of cpDNA and nrDNA. Aust. Syst. Bot. 28, 111–123. 10.1071/SB15008

[B14] Bergman J. Lindström J.-O. Tilstam U. (1985). The structure and properties of some indolic constituents in *Couroupita guianensis aubl* . Tetrahedron 41, 2879–2881. 10.1016/S0040-4020(01)96609-8

[B15] Bharathi Dileepan A. G. Daniel Prakash T. Ganesh Kumar A. Shameela Rajam P. Violet Dhayabaran V. Rajaram R. (2018). Isatin based macrocyclic Schiff base ligands as novel candidates for antimicrobial and antioxidant drug design: *in vitro* DNA binding and biological studies. J. Photochem. Photobiol. B Biol. 183, 191–200. 10.1016/j.jphotobiol.2018.04.029 29723731

[B16] Borges B. E. Teixeira V. R. Appel M. H. Steclan C. A. Rigo F. Filipak Neto F. (2013). De novo galectin-3 expression influences the response of melanoma cells to isatin-Schiff base copper(II) complex-induced oxidative stimulus. Chem. Biol. Interact. 206, 37–46. 10.1016/j.cbi.2013.08.005 23994248

[B17] Bramson H. N. Corona J. Davis S. T. Dickerson S. H. Edelstein M. Frye S. V. (2001). Oxindole-based inhibitors of cyclin-dependent kinase 2 (CDK2): Design, synthesis, enzymatic activities, and X-ray crystallographic analysis. J. Med. Chem. 44, 4339–4358. 10.1021/jm010117d 11728181

[B18] Castelli S. Gonçalves M. B. Katkar P. Stuchi G. C. Couto R. A. A. Petrilli H. M. (2018). Comparative studies of oxindolimine-metal complexes as inhibitors of human DNA topoisomerase IB. J. Inorg. Biochem. 186, 85–94. 10.1016/j.jinorgbio.2018.05.012 29860208

[B19] Cerchiaro G. Aquilano K. Filomeni G. Rotilio G. Ciriolo M. R. Ferreira A. M. (2005). Isatin-Schiff base copper(II) complexes and their influence on cellular viability. J. Inorg. Biochem. 99, 1433–1440. 10.1016/j.jinorgbio.2005.03.013 15878622

[B20] Cerchiaro G. Ferreira A. M. D. C. (2006). Oxindoles and copper complexes with oxindole-derivatives as potential pharmacological agents. J. Braz. Chem. Soc. 17, 1473–1485. 10.1590/S0103-50532006000800003

[B21] Champoux J. J. (2001). DNA topoisomerases: structure, function, and mechanism. Annu. Rev. Biochem. 70, 369–413. 10.1146/annurev.biochem.70.1.369 11395412

[B130] Cheke R.S. Firke S.D. Patila R.R. Bari S.B. (2018). ISATIN: new hope against convulsion. Central Nervous System Agents Med. Chem. 18, 76–101. 10.2174/1871524917666171113124112 29141569

[B22] Choi S. J. Lee J. E. Jeong S. Y. Im I. Lee S. D. Lee E. J. (2010). 5,5′-substituted indirubin-3′-oxime derivatives as potent cyclin-dependent kinase inhibitors with anticancer activity. J. Med. Chem. 53, 3696–3706. 10.1021/jm100080z 20361800

[B23] Crown J. P. Diéras V. Staroslawska E. Yardley D. A. Bachelot T. Davidson N. (2013). Phase III trial of sunitinib in combination with capecitabine versus capecitabine monotherapy for the treatment of patients with pretreated metastatic breast cancer. J. Clin. Oncol. 31, 2870–2878. 10.1200/JCO.2012.43.3391 23857972

[B24] Da Silva J. F. M. Garden S. J. Pinto A. C. (2001). The Chemistry of Isatins: A Review from 1975 to 1999. J. Braz. Chem. Soc. 12, 273–324. 10.1590/s0103-50532001000300002

[B25] Da Silveira V. C. Luz J. S. Oliveira C. C. Graziani I. Ciriolo M. R. da Costa Ferreira A. M. (2008). Double-strand DNA cleavage induced by oxindole-Schiff base copper(II) complexes with potential antitumor activity. J. Inorg. Biochem. 102, 1090–1103. 10.1016/j.jinorgbio.2007.12.033 18295339

[B26] Dario B. S. Fernandes Neto F. Portes M. C. Boni Fazzi R. Rodrigues da Silva D. Peterson E. J. (2019). DNA binding, cytotoxic effects and probable targets of an oxindolimine–vanadyl complex as an antitumor agent. New J. Chem. 43, 17831–17840. 10.1039/C9NJ02480H

[B27] Davidovich P. Aksenova V. Petrova V. Tentler D. Orlova D. Smirnov S. (2015). Discovery of novel isatin-based p53 inducers. ACS Med. Chem. Lett. 6, 856–860. 10.1021/acsmedchemlett.5b00011 26288684 PMC4538431

[B28] Hoog P. Boldron C. Gamez P. Sliedregt-Bol K. Roland I. Pitié M. (2007). New approach for the preparation of efficient DNA cleaving agents: ditopic copper-platinum complexes based on 3-clip-phen and cisplatin. J. Med. Chem. 50, 3148–3152. 10.1021/jm0614331 17521178

[B29] Ding Z. Zhou M. Zeng C. (2020). Recent advances in isatin hybrids as potential anticancer agents. Arch. Pharm. 353 (3), 1900367 10.1002/ardp.201900367 31960987

[B30] Dweedar H. E. Mahrous H. Ibrahim H. S. Abdel-Aziz H. A. (2014). Analogue-based design, synthesis and biological evaluation of 3-substituted-(methylenehydrazono)indolin-2-ones as anticancer agents. Eur. J. Med. Chem. 78, 275–280. 10.1016/j.ejmech.2014.03.058 24686014

[B31] Eisen T. Loembé A. B. Shparyk Y. Macleod N. Jones R. J. Mazurkiewicz M. (2015). A randomised, phase II study of nintedanib or sunitinib in previously untreated patients with advanced renal cell cancer: 3-year results. Br. J. Cancer 113, 1140–1147. 10.1038/bjc.2015.313 26448178 PMC4647871

[B32] El-Faham A. Farooq M. Khattab S. N. Abutaha N. Wadaan M. A. Ghabbour H. A. (2015). Synthesis, characterization, and anti-cancer activity of some new N′-(2-Oxoindolin-3-ylidene)-2-propylpentane hydrazide-hydrazones derivatives. Molecules 20, 14638–14655. 10.3390/molecules200814638 26287132 PMC6332339

[B33] Eldehna W. M. Abo-Ashour M. F. Ibrahim H. S. Al-Ansary G. H. Ghabbour H. A. Elaasser M. M. (2018). Novel [(3-indolylmethylene)hydrazono]indolin-2-ones as apoptotic anti-proliferative agents: design, synthesis and *in vitro* biological evaluation. J. Enzyme Inhib. Med. Chem. 33, 686–700. 10.1080/14756366.2017.1421181 29560733 PMC6010103

[B34] Eldehna W. M. Almahli H. Al-Ansary G. H. Ghabbour H. A. Aly M. H. Ismael O. E. (2017). Synthesis and in vitro anti-proliferative activity of some novel isatins conjugated with quinazoline/phthalazine hydrazines against triple-negative breast cancer MDA-MB-231 cells as apoptosis-inducing agents. J. Enzyme Inhib. Med. Chem. 32, 600–613. 10.1080/14756366.2017.1279155 28173708 PMC6010087

[B35] Eldehna WM Altoukhy A Mahrous H Abdel-Aziz HA (2015). Design, synthesis and QSAR study of certain isatin-pyridine hybrids as potential anti-proliferative agents. Eur. J. Med. Chem. 90, 684–694. 10.1016/j.ejmech.2014.12.010 25499988

[B36] Elder D. P. Snodin D. Teasdale A. (2011). Control and analysis of hydrazine, hydrazides and hydrazones--genotoxic impurities in active pharmaceutical ingredients (APIs) and drug products. J. Pharm. Biomed. Anal. 54, 900–910. 10.1016/j.jpba.2010.11.007 21145684

[B37] Esmaeelian B. Abbott C. A. Le Leu R. K. Benkendorff K. (2014). 6-Bromoisatin found in muricid mollusc extracts inhibits colon cancer cell proliferation and induces apoptosis, preventing early stage tumor formation in a colorectal cancer rodent model. Mar. Drugs 12, 17–35. 10.3390/md12010017 PMC3917258 24368567

[B38] Evdokimov N. M. Magedov I. V. McBrayer D. Kornienko A. (2016). Isatin derivatives with activity against apoptosis-resistant cancer cells. Bioorg. Med. Chem. Lett. 26, 1558–1560. 10.1016/j.bmcl.2016.02.015 26883150 PMC4775416

[B39] Fares M. Eldehna W. M. Abou-Seri S. M. Abdel-Aziz H. A. Aly M. H. Tolba M. F. (2015). Design, synthesis and *in vitro* antiproliferative activity of novel isatin-quinazoline hybrids. Arch Pharm. 348, 144–154. 10.1002/ardp.201400337 25664631

[B40] Farooq M. Al Marhoon Z. M. Taha N. A. Baabbad A. A. Al-Wadaan M. A. El-Faham A. (2018). Synthesis of novel class of n-alkyl-isatin-3-iminobenzoic acid derivatives and their biological activity in zebrafish embryos and human cancer cell lines. Biol. Pharm. Bull. 41, 350–359. 10.1248/bpb.b17-00674 29249771

[B41] Filomeni G. Cerchiaro G. Da Costa Ferreira A. M. De Martino A. Pedersen J. Z. Rotilio G. (2007). Pro-apoptotic activity of novel isatin-schiff base copper(II) complexes depends on oxidative stress induction and organelle-selective damage. J. Biol. Chem. 282, 12010–12021. 10.1074/jbc.M610927200 17327230

[B42] Filomeni G. Cardaci S. Da Costa Ferreira A. M. Rotilio G. Ciriolo M. R. (2011). Metabolic oxidative stress elicited by the copper(II) complex [Cu(isaepy)2] triggers apoptosis in SH-SY5Y cells through the induction of the AMP-activated protein kinase/p38MAPK/p53 signalling axis: evidence for a combined use with 3-bromopyruvate in neuroblastoma treatment. Biochem. J. 437, 443–453. 10.1042/BJ20110510 21548882

[B43] Filomeni G. Piccirillo S. Graziani I. Cardaci S. Da Costa Ferreira A. M. Rotilio G. (2009). The isatin-Schiff base copper(II) complex Cu(isaepy)2 acts as delocalized lipophilic cation, yields widespread mitochondrial oxidative damage and induces AMP-activated protein kinase-dependent apoptosis. Carcinogenesis 30, 1115–1124. 10.1093/carcin/bgp105 19406932 PMC2722147

[B44] Gou Y. Li J. Fan B. Xu B. Zhou M. Yang F. (2017). Structure and biological properties of mixed-ligand Cu(II) schiff base complexes as potential anticancer agents. Eur. J. Med. Chem. 134, 207–217. 10.1016/j.ejmech.2017.04.026 28415010

[B45] Guo H. (2019). Isatin derivatives and their anti-bacterial activities. Eur. J. Med. Chem. 164, 678–688. 10.1016/j.ejmech.2018.12.017 30654239

[B46] Hall M. D. Salam N. K. Hellawell J. L. Fales H. M. Kensler C. B. Ludwig J. A. (2009). Synthesis, activity, and pharmacophore development for isatin-beta-thiosemicarbazones with selective activity toward multidrug-resistant cells. J. Med. Chem. 52, 3191–3204. 10.1021/jm800861c 19397322 PMC2744114

[B47] Hall M. D. Brimacombe K. R. Varonka M. S. Pluchino K. M. Monda J. K. Li J. (2011). Synthesis and structure-activity evaluation of isatin-β-thiosemicarbazones with improved selective activity toward multidrug-resistant cells expressing P-glycoprotein. J. Med. Chem. 54, 5878–5889. 10.1021/jm2006047 21721528 PMC3201829

[B48] Hamama W. S. El-Bana G. G. Shaaban S. Zoorob H. H. (2018). Synthetic approach to some new annulated 1,2,4-triazine skeletons with antimicrobial and cytotoxic activities. J. Heterocycl. Chem. 55, 971–982. 10.1002/jhet.3127

[B49] Haribabu J. Jeyalakshmi K. Arun Y. Bhuvanesh N. S. P. Perumal P. T. Karvembu R. (2015). Synthesis, DNA/protein binding, molecular docking, DNA cleavage and in vitro anticancer activity of nickel(II) bis(thiosemicarbazone) complexes. RSC Adv 5, 46031–46049. 10.1039/c5ra04498g

[B50] Harris P. (2007). Oxindole inhibitors of cyclin-dependent kinases as anti-tumor agents, chap. 12. Boca Raton, FL: CRC Press, Taylor & Francis, 265–281. 10.1201/9781420005400.ch12

[B51] He J. Xiao H. Li B. Peng Y. Li X. Wang Y. (2019). The programmed site-specific delivery of the angiostatin sunitinib and chemotherapeutic paclitaxel for highly efficient tumor treatment. J. Mater. Chem. B 7, 4953–4962. 10.1039/C9TB01159E 31411627

[B52] Hunoor R. S. Patil B. R. Badiger D. S. Chandrashekhar V. M. Muchchandi I. S. Gudasi K. B. (2015). Co(II), Ni(II), Cu(II) and Zn(II) complexes of isatinyl-2-aminobenzoylhydrazone: synthesis, characterization and anticancer activity. Appl. Organomet. Chem. 29, 101–108. 10.1002/aoc.3252

[B53] Ibrahim H. S. Abou-Seri S. M. Ismail N. S. M. Elaasser M. M. Aly M. H. Abdel-Aziz H. A. (2016). Bis-isatin hydrazones with novel linkers: Synthesis and biological evaluation as cytotoxic agents. Eur. J. Med. Chem. 108, 415–422. 10.1016/j.ejmech.2015.11.047 26706352

[B54] Ismail M. F. El-sayed A. A. (2019). Synthesis and in-vitro antioxidant and antitumor evaluation of novel pyrazole-based heterocycles. J. Iran. Chem. Soc. 16, 921–937. 10.1007/s13738-018-1566-x

[B55] Izzedine H. Buhaescu I. Rixe O. Deray G. (2007). Sunitinib malate. Cancer Chemother. Pharmacol 60, 357–364. 10.1007/s00280-006-0376-5 17136543

[B131] Kajal A. Bala S. Kamboj S. Sharma N. Saini V. (2013). Schiff bases: a versatile pharmacophore. J. Catalysts 2013, 893512 10.1155/2013/893512

[B56] Kanai F. Yoshida H. Tateishi R. Sato S. Kawabe T. Obi S. (2011). A phase I/II trial of the oral antiangiogenic agent TSU-68 in patients with advanced hepatocellular carcinoma. Cancer Chemother. Pharmacol. 67, 315–324. 10.1007/s00280-010-1320-2 20390419

[B57] Kandile N. G. Mohamed M. I. Ismaeel H. M. (2012). Antiproliferative effects of metal complexes of new isatin hydrazones against HCT116, MCF7 and HELA tumour cell lines. J. Enzyme Inhib. Med. Chem. 27, 330–338. 10.3109/14756366.2011.588950 21699460

[B58] Karnthaler-Benbakka C. Koblmüller B. Mathuber M. Holste K. Berger W. Heffeter P. (2019). Synthesis, characterization and *in vitro* studies of a cathepsin B-cleavable prodrug of the VEGFR inhibitor sunitinib. Chem. Biodivers. 16, e1800520 10.1002/cbdv.201800520 30566287 PMC6391952

[B59] Katkar P. Coletta A. Castelli S. Sabino G. L. Couto R. A. Ferreira A. M. (2014). Effect of oxindolimine copper(II) and zinc(II) complexes on human topoisomerase I activity. Metallomics 6, 117–125. 10.1039/C3MT00099K 24172750

[B60] Kaur M. Singh M. Chadha N. Silakari O. (2016). Oxindole: a chemical prism carrying plethora of therapeutic benefits. Eur. J. Med. Chem. 123, 858–894. Elsevier Ltd. 10.1016/j.ejmech.2016.08.011 27543880

[B61] Kerzarea D. R. Khedekar P. B. (2016). Indole derivatives acting on central nervous system—review. J Pharm Sci Biosci. Res. 6, 144–156

[B62] Kudo M. Cheng A. L. Park J. W. Park J. H. Liang P. C. Hidaka H. (2018). Orantinib versus placebo combined with transcatheter arterial chemoembolisation in patients with unresectable hepatocellular carcinoma (ORIENTAL): a randomised, double-blind, placebo-controlled, multicentre, phase 3 study. Lancet Gastroenterol. Hepatol. 3, 37–46. 10.1016/S2468-1253(17)30290-X 28988687

[B63] Kumar M. R. Dhayabaran V. Sudhapriya N. Manikandan A. Gideon D. A. Annapoorani S. (2020). p-TSA.H2O mediated one-pot, multi-component synthesis of isatin derived imidazoles as dual-purpose drugs against inflammation and cancer. Bioorg. Chem. 102, 104046 10.1016/j.bioorg.2020.104046 32688115

[B64] Lane M. E. Yu B. Rice A. Lipson K. E. Liang C. Sun L. (2001). A novel CDK2-selective inhibitor, SU9516, induces apoptosis in colon carcinoma cells 1. Cancer Res, 61, 6170 11507069

[B65] Lawrence H. R. Pireddu R. Chen L. Luo Y. Sung S. S. Szymanski A. M. (2008). Inhibitors of Src homology-2 domain containing protein tyrosine phosphatase-2 (Shp2) based on oxindole scaffolds. J. Med. Chem. 51, 4948–4956. 10.1021/jm8002526 18680359 PMC2744494

[B66] Le Goff G. Ouazzani J. (2014). Natural hydrazine-containing compounds: biosynthesis, isolation, biological activities and synthesis. Bioorganic Med. Chem. 22, 6529–6544. 10.1016/j.bmc.2014.10.011 25456382

[B67] Le Tourneau C. Raymond E. Faivre S. (2007). Sunitinib: A novel tyrosine kinase inhibitor. a brief review of its therapeutic potential in the treatment of renal carcinoma and gastrointestinal stromal tumors (GIST). Ther. Clin. Risk Manag. 3, 341–348. 10.2147/tcrm.2007.3.2.341 18360643 PMC1936316

[B68] Liang C. Xia J. Lei D. Li X. Yao Q. Gao J. (2014). Synthesis, *in vitro* and *in vivo* antitumor activity of symmetrical bis-schiff base derivatives of isatin. Eur. J. Med. Chem. 74, 742–750. 10.1016/j.ejmech.2013.04.040 24176732

[B69] Lockhart A. C. Cropp G. F. Berlin J. D. Donnelly E. Schumaker R. D. Schaaf L. J. (2006). Phase I/Pilot study of SU5416 (Semaxinib) in combination with Irinotecan/Bolus 5-FU/LV (IFL) in patients with metastatic colorectal cancer. Am. J. Clin. Oncol. 29, 109–15. 10.1097/01.coc.0000199882.53545.ac 16601426

[B70] London C. A. Hannah A. L. Zadovoskaya R. Chien M. B. Kollias-Baker C. Rosenberg M. (2003). Phase I dose-escalating study of SU11654, a small molecule receptor tyrosine kinase inhibitor, in dogs with spontaneous malignancies. Clin. Cancer Res. 9, 2755–2768. Available at: http://www.ncbi.nlm.nih.gov/pubmed/12855656 12855656

[B71] London C. A. Malpas P. B. Wood-Follis S. L. Boucher J. F. Rusk A. W. Rosenberg M. P. (2009). Multi-center, placebo-controlled, double-blind, randomized study of oral toceranib phosphate (SU11654), a receptor tyrosine kinase inhibitor, for the treatment of dogs with recurrent (either local or distant) mast cell tumor following surgical excision. Clin. Cancer Res. 15, 3856–3865. 10.1158/1078-0432.CCR-08-1860 19470739

[B72] Mamián-López M. B. Miguel R. B. Araki K. A. Temperini M. L. Da Costa Ferreira A. M. (2021). Multivariate probing of antitumor metal-based complexes damage on living cells through Raman imaging. Spectrochim. Acta Part A Mol. Biomol. Spectrosc. 244, 118838 10.1016/j.saa.2020.118838 32862078

[B73] Marchese A. D. Larin E. M. Mirabi B. Lautens M. (2020). Metal-catalyzed approaches toward the oxindole core. Acc. Chem. Res. 53 (8), 1605–1619. 10.1021/acs.accounts.0c00297 32706589

[B74] Marques C. S. López Ó. Bagetta D. Carreiro E. P. Petralla S. Bartolini M. (2020). N-1,2,3-triazole-isatin derivatives for cholinesterase and β-amyloid aggregation inhibition: a comprehensive bioassay study. Bioorg. Chem. 98, 103753 10.1016/j.bioorg.2020.103753 32200328

[B75] McCormack P. L. (2015). Nintedanib: first global approval. Drugs 75, 129–139. 10.1007/s40265-014-0335-0 25430078

[B76] Medvedev A. Igosheva N. Crumeyrolle-Arias M. Glover V. (2005). Isatin: role in stress and anxiety. Stress 8, 175–183. 10.1080/10253890500342321 16236622

[B77] Miguel R. B. Petersen P. A. Gonzales-Zubiate F. A. Oliveira C. C. Kumar N. do Nascimento R. R. (2015). Inhibition of cyclin-dependent kinase CDK1 by oxindolimine ligands and corresponding copper and zinc complexes. J. Biol. Inorg. Chem. 20, 1205–1217. 10.1007/s00775-015-1300-4 26411703

[B78] Nam N. H. Huong T. L. Dung D. T. Dung P. T. Oanh D. T. Quyen D. (2013). Novel isatin-based hydroxamic acids as histone deacetylase inhibitors and antitumor agents. Eur. J. Med. Chem. 70, 477–486. 10.1016/j.ejmech.2013.10.045 24185378

[B79] Nasr T. Bondock S. Youns M. (2014). Anticancer activity of new coumarin substituted hydrazide-hydrazone derivatives. Eur. J. Med. Chem. 76, 539–548. 10.1016/j.ejmech.2014.02.026 24607878

[B80] Nath P. Mukherjee A. Mukherjee S. Banerjee S. Das S. Banerjee S. (2020). Isatin: a scaffold with innumerable biodiversity. Mini Rev. Med. Chem. [E-pub ahead of print]. 10.2174/2211536609666201125115559 33238872

[B81] Naz F Anjum F Islam A Ahmad F Hassan MI (2013). Microtubule Affinity-Regulating Kinase 4: Structure, Function, and Regulation. Cell Biochem. Biophys. 67, 485–499. 10.1007/s12013-013-9550-7 23471664

[B82] Nicholas J. Said M. Harshani R. Moffitt H. L. (2015). Data, R. U.S.A.. (12) United States Patent. 2

[B83] Osman S. A. Mousa H. A. Yosef H. A. A. Hafez T. S. El-Sawy A. A. Abdallah M. M. (2014). Synthesis, characterization and cytotoxicity of mixed ligand Mn(II), Co(II) and Ni(II) complexes. J. Serbian Chem. Soc. 79, 953–964. 10.2298/JSC130813134O

[B84] Pacheco de Oliveira H. (2011). Lecythidaceae—*Couroupita guianensis aubl* . Available at: https://commons.wikimedia.org/w/index.php?curid=17564165

[B85] Pandeya S. N. Smitha S. Jyoti M. Sridhar S. K. (2005). Biological activities of isatin and its derivatives. Acta Pharm 55, 27–46 15907222

[B86] Pati M. L. Niso M. Ferorelli S. Abate C. Berardi F. (2015). Novel metal chelators thiosemicarbazones with activity at the σ2 receptors and P-glycoprotein: An innovative strategy for resistant tumor treatment. RSC Adv. 5, 103131–103146. 10.1039/c5ra19857g

[B87] Pati M. L. Niso M. Spitzer D. Berardi F. Contino M. Riganti C. (2018). Multifunctional thiosemicarbazones and deconstructed analogues as a strategy to study the involvement of metal chelation, sigma-2 (σ2) receptor and P-gp protein in the cytotoxic action: In vitro and in vivo activity in pancreatic tumors. Eur. J. Med. Chem. 144, 359–371. 10.1016/j.ejmech.2017.12.024 29287249 PMC5801006

[B88] Patra J. K. Das G. Fraceto L. F. Campos E. V. R. Rodriguez-Torres M. D. P. Acosta-Torres L. S. (2018). Nano based drug delivery systems: recent developments and future prospects. J. Nanobiotechnology 16, 71 10.1186/s12951-018-0392-8 30231877 PMC6145203

[B89] Pommier Y. (2006). Topoisomerase I inhibitors: camptothecins and beyond. Nat. Rev. Cancer 6, 789–802. 10.1038/nrc1977 16990856

[B90] Prakash B. Amuthavalli A. Edison D. Sivaramkumar M. S. Velmurugan R. (2018). Novel indole derivatives as potential anticancer agents: design, synthesis and biological screening. Med. Chem. Res. 27, 321–331. 10.1007/s00044-017-2065-9

[B91] Prakash C. R. Theivendren P. Raja S. (2012). Indolin-2-ones in clinical trials as potential kinase inhibitors: a review. Pharmacol. and Pharm. 3, 62–71. 10.4236/pp.2012.31010

[B92] Puliyappadamba V. T. Wu W. Bevis D. Zhang L. Polin L. Kilkuskie R. (2011). Antagonists of anaphase-promoting complex (APC)-2-cell cycle and apoptosis regulatory protein (CARP)-1 interaction are novel regulators of cell growth and apoptosis. J. Biol. Chem. 286, 38000–38017. 10.1074/jbc.M111.222398 21903591 PMC3207393

[B93] Rahim F. Malik F. Ullah H. Wadood A. Khan F. Javid M. T. (2015). Isatin based schiff bases as inhibitors of α-glucosidase: synthesis, characterization, *in vitro* evaluation and molecular docking studies. Bioorg. Chem. 60, 42–48. 10.1016/j.bioorg.2015.03.005 25955493

[B94] Raj R. Gut J. Rosenthal P. J. Kumar V. (2014). 1H-1,2,3-triazole-tethered isatin-7-chloroquinoline and 3-hydroxy-indole-7-chloroquinoline conjugates: synthesis and antimalarial evaluation. Bioorg Med Chem Lett 24, 756–759. 10.1016/j.bmcl.2013.12.109 24424135

[B95] Rios R. (2012). Enantioselective methodologies for the synthesis of spiro compounds. Chem. Soc. Rev. 41, 1060–1074. 10.1039/c1cs15156h 21975423

[B96] Rizzo M. Porta C. (2017). Sunitinib in the treatment of renal cell carcinoma: An update on recent evidence. Ther. Adv. Urol. 9, 195–207. 10.1177/1756287217713902 29662544 PMC5896861

[B97] Robert N. J. Saleh M. N. Paul D. Generali D. Gressot L. Copur M. S. (2011). Sunitinib plus paclitaxel versus bevacizumab plus paclitaxel for first-line treatment of patients with advanced breast cancer: a phase III, randomized, open-label trial. Clin. Breast Cancer 11, 82–92. 10.1016/j.clbc.2011.03.005 21569994 PMC4617186

[B98] Rodríguez-Argüelles M. C. Sánchez A. Belicchi Ferrari M. Gasparri Fava G. Pelizzi C. Pelosi G. (1999). Transition-metal complexes of isatin-P-thiosemicarbazone. X-ray crystal structure of two nickel complexes. J. Inorg. Biochem. 73 (1–2), 7–15. 10.1016/s0162-0134(98)10085-5 10212992

[B99] Rodríguez-Argüelles M. C. Cao R. García-Deibe A. M. Pelizzi C. Sanmartín-Matalobos J. Zani F. (2009). Antibacterial and antifungal activity of metal(II) complexes of acylhydrazones of 3-isatin and 3-(N-methyl)isatin. Polyhedron 28, 2187–2195. 10.1016/j.poly.2008.12.038

[B100] Roskoski R. (2007). Sunitinib: a VEGF and PDGF receptor protein kinase and angiogenesis inhibitor. Biochem. Biophys. Res. Commun. 356, 323–328. 10.1016/j.bbrc.2007.02.156 17367763

[B132] Saini T. Kumar S. Narasimhan B. (2015). Central nervous system activities of Indole derivatives: an overview. Central Nervous System Agents Med. Chem. 16, 19–28. 10.2174/1871524915666150608103224 26051466

[B101] Scott L. M. Lawrence H. R. Sebti S. M. Lawrence N. J. Wu J. (2010). Targeting protein tyrosine phosphatases for anticancer drug discovery. Curr. Pharm. Des. 16, 1843–1862. 10.2174/138161210791209027 20337577 PMC3076191

[B102] Silva B. V. (2013). Isatin, a versatile molecule: studies in Brazil. J. Braz. Chem. Soc. 24, 707–720. 10.5935/0103-5053.20130089

[B103] Singh A. P. Biswas A. Shukla A. Maiti P. (2019). Targeted therapy in chronic diseases using nanomaterial-based drug delivery vehicles. Signal Transduct. Target. Ther. 4, 33 10.1038/s41392-019-0068-3 31637012 PMC6799838

[B104] Singh G. S. Desta Z. Y. (2012). Isatins as privileged molecules in design and synthesis of spiro-fused cyclic frameworks. Chem. Rev. 112, 6104–61 55. 10.1021/cr300135y 22950860

[B105] Singh H. Singh J. V. Gupta M. K. Saxena A. K. Sharma S. Nepali K. (2017). Triazole tethered isatin-coumarin based molecular hybrids as novel antitubulin agents: Design, synthesis, biological investigation and docking studies. Bioorg Med Chem Lett 27, 3974–3979. 10.1016/j.bmcl.2017.07.069 28797799

[B106] Singh N. K. Kumbhar A. A. Pokharel Y. R. Yadav P. N. (2020). Anticancer potency of copper(II) complexes of thiosemicarbazones. J. Inorg. Biochem. 210, 111134 10.1016/j.jinorgbio.2020.111134 32673842

[B107] Spencer J. Amin J. Callear S. K. Tizzard G. J. Coles S. J. Coxhead P. (2011a). Synthesis and evaluation of metallocene containing methylidene-1,3-dihydro-2H-indol-2-ones as kinase inhibitors. Metallomics 3, 600–608. 10.1039/c1mt00017a 21359402

[B108] Spencer J. Amin J. Coxhead P. McGeehan J. Richards C. J. Tizzard G. J. (2011b). Size does matter. Sterically demanding metallocene-substituted 3-methylidene-oxindoles exhibit poor kinase inhibitory action. Organometallics 30, 3177–3181. 10.1021/om200278j

[B109] Sridhar S. K. Pandeya S. N. Stables J. P. Ramesh A. (2002). Anticonvulsant activity of hydrazones, schiff and mannich bases of isatin derivatives. Eur. J. Pharm. Sci. 16, 129–132. 10.1016/S0928-0987(02)00077-5 12128166

[B110] Tadele K. T. Tsega T. W. (2019). Schiff bases and their metal complexes as potential anticancer candidates: a review of recent works. Anticancer. Agents Med. Chem. 19, 1786–1795. 10.2174/1871520619666190227171716 30827264

[B111] Thomas A. Pommier Y. (2019). Targeting topoisomerase I in the era of precision medicine. Clin. Cancer Res. 25, 6581–6589. 10.1158/1078-0432.CCR-19-1089 31227499 PMC6858945

[B112] Van Cutsem E. Yoshino T. Lenz H. J. Lonardi S. Falcone A. Limón M. L. (2018). Nintedanib for the treatment of patients with refractory metastatic colorectal cancer (LUME-Colon 1): a phase III, international, randomized, placebo-controlled study. Ann. Oncol. 29, 1955–1963. 10.1093/annonc/mdy241 30010751 PMC6158765

[B113] Varun V. Sonam S. Kakkar R. (2019). Isatin and its derivatives: a survey of recent syntheses, reactions, and applications. Medchemcomm 10, 351–368. 10.1039/C8MD00585K 30996856 PMC6438150

[B114] Vieira E. G. Miguel R. B. da Silva D. R. Fazzi R. B. de Couto R. A. A. Marin J. H. (2019). Functionalized nanoparticles as adjuvant to increase the cytotoxicity of metallodrugs toward tumor cells. New J. Chem. 43, 386–398. 10.1039/C8NJ04654A

[B115] Vigato P. A. Tamburini S. (2004). The challenge of cyclic and acyclic schiff bases and related derivatives. Coord. Chem. Rev. 248, 1717–2128. 10.1016/j.ccr.2003.09.003

[B116] Vigato P. A. Tamburini S. Bertolo L. (2007). The development of compartmental macrocyclic schiff bases and related polyamine derivatives. Coord. Chem. Rev. 251, 1311–1492. 10.1016/j.ccr.2006.11.016

[B117] Vine K. Matesic L. Locke J. Ranson M. Skropeta D. (2009). Cytotoxic and anticancer activities of isatin and its derivatives: a comprehensive review from 2000–2008. Anticancer. Agents Med. Chem. 9, 397–414. 10.2174/1871520610909040397 19442041

[B118] Vine K. L. Matesic L. Locke J. M. Skropeta D. (2013). Recent highlights in the development of isatin-based anticancer agents. Adv. Anticancer Agents Med. Chem. 2, 254–312. 10.2174/9781608054961113020008

[B119] Vintonyak V. V. Warburg K. Kruse H. Grimme S. Hübel K. Rauh D. (2010). Identification of thiazolidinones spiro-fused to indolin-2-ones as potent and selective inhibitors of the *Mycobacterium tuberculosis* protein tyrosine phosphatase B. Angew Chem Int Ed Engl 49, 5902–5905. 10.1002/anie.201002138 20632348

[B120] Xu Z. Zhang S. Gao C. Fan J. Zhao F. Lv Z. S. (2017). Isatin hybrids and their anti-tuberculosis activity. Chinese Chem. Lett. 28, 159–167. 10.1016/j.cclet.2016.07.032

[B121] Yang Y. Bu P. (2016). Progress on the cardiotoxicity of sunitinib: Prognostic significance, mechanism and protective therapies. Chem. Biol. Interact. 257, 125–131. 10.1016/j.cbi.2016.08.006 27531228

[B122] Yang M. Liu H. Zhang Y. Wang X. Zhi Xu Z. (2020). Moxifloxacin-isatin hybrids tethered by 1,2,3-triazole and their anticancer activities. Curr. Topics Med. Chem. 20, 1461–1467. 10.2174/1568026620666200128144825 31994464

[B123] Ye N. Chen H. Wold E. A. Shi P. Y. Zhou J. (2016). Therapeutic potential of spirooxindoles as antiviral agents. ACS Infect. Dis. 2, 382–392. 10.1021/acsinfecdis.6b00041 27627626 PMC5417367

[B124] Yekke-Ghasemi Z. Ramezani M. Mague J. T. Takjoo R. (2020). Synthesis, characterization and bioactivity studies of new dithiocarbazate complexes. New J. Chem. 44, 8878–8889. 10.1039/d0nj01187h

[B125] Yongvongsoontorn N. Chung J. E. Gao S. J. Bae K. H. Yamashita A. Tan M. H. (2019). Carrier-enhanced anticancer efficacy of sunitinib-loaded green tea-based micellar nanocomplex beyond tumor-targeted delivery. ACS Nano 13, 7591–7602. 10.1021/acsnano.9b00467 31262169

[B126] Yousef M. A. Ali A. M. El-Sayed W. M. Qayed W. S. Farag H. H. A. Aboul-Fadl T. (2020). Design and synthesis of novel isatin-based derivatives targeting cell cycle checkpoint pathways as potential anticancer agents. Bioorg. Chem. 105, 104366 10.1016/j.bioorg.2020.104366 33212312

[B127] Zhang M. Z. Chen Q. Yang G. F. (2015). A review on recent developments of indole-containing antiviral agents. Eur. J. Med. Chem. 89, 421–441. 10.1016/j.ejmech.2014.10.065 25462257 PMC7115707

[B128] Zhou J. Qu F. (2011). Analysis of the extracts of Isatis tinctoria by new analytical approaches of HPLC, MS and NMR. Afr J Tradit Complement Altern Med 8, 33–45. 10.4314/ajtcam.v8i5S.13 22754056 PMC3252730

[B129] Zhu J. Lin M. Fan D. Wu Z. Chen Y. Zhang J. (2009). The role of bridging ligands in determining DNA-binding ability and cross-linking patterns of dinuclear platinum(II) antitumour complexes. Dalton Trans., 10889–95. 10.1039/b913236h 20023919

