# Supplementary material for: Anticancer Compounds Based on Isatin-Derivatives: Strategies to Ameliorate Selectivity and Efficiency
Source: Front Mol Biosci. 2021 Feb 4;7:627272. doi: 10.3389/fmolb.2020.627272 (PMC7889591; doi:10.3389/fmolb.2020.627272)
Supplement: Supplementary file 1 [file datasheet1.docx]

**Table S1**. Summary of kinase inhibition potencies exhibited by metallocenes functionalized with oxindole motifs (Spencer et al., 2011a, 2011b)**.** PAK1, p21-activated kinase-1; VEGFR-2, Vascular endothelial growth factor receptor 2; DYRK, dual specificity tyrosine-phosphorylation-regulated kinase (isoforms 1a, 2, 3 and 4).

| **Compound** | **PAK1 (% residual activity)** | **VEGFR2 IC_50_ (μM)** | **DYRK1a IC_50_ (μM)** | **DYRK2 IC_50_ (μM)** | **DYRK3 IC_50_ (μM)** | **DYRK4 IC_50_ (μM)** |
| --- | --- | --- | --- | --- | --- | --- |
| *Sunitinib* | --- | 0.0015 | --- | --- | --- | --- |
| *E*-A | 100 | *0.21* | *i* | *i* | >10 | 0.47 |
| *Z*-A | 100 | *0.22* | *i* | *i* | 0.39 | 1.1 |
| *E*-B | 88 | *---* | --- | --- | n.d. | n.d. |
| *Z*-B | 93 | *---* | --- | --- | >10 | >10 |
| *E*-C | 100 | *---* | --- | --- | *i* | *i* |
| *Z*-C | 83 | *---* | --- | --- | *i* | *i* |
| *Z-D* | --- | *i* | *i* | *i* | 1.1 | 4.5 |
| *E-*E | --- | *i* | *i* | *i* | *i* | 1.5 |
| *Z-*E | --- | *i* | *i* | *i* | 0.96 | 0.65 |
| *E-F* | --- | *1.3* | *i* | *i* | *i* | *i* |
| *Z-F* | --- | *1.9* | *i* | *i* | *i* | *i* |
| *Z-G* | --- | *0.12* | *i* | 1.1 | 0.9 | 2.7 |
| *Z-H* | --- | 1.3 | 6.8 | 12 | 2 | 1.9 |

*i = inactive; --- = no data available.*
